# Prognostic Value of Regression Rate of Plasma EBV DNA After Induction Chemotherapy for Stage II-IVA Nasopharyngeal Carcinoma

**DOI:** 10.3389/fonc.2021.689593

**Published:** 2021-07-15

**Authors:** Hao Peng, Bin-bin Chen, Xiao-hui Wang, Yun-Xian Mo, Fei Han

**Affiliations:** ^1^ Center for Translational Medicine, Precision Medicine Institute, The First Affiliated Hospital, Sun Yat-sen University, Guangzhou, China; ^2^ Department of Radiation Oncology, Sun Yat-sen University Cancer Center, Guangzhou, China; ^3^ Department of Pediatric Oncology, Sun Yat-sen University Cancer Center, Guangzhou, China; ^4^ State Key Laboratory of Oncology in South China, Collaborative Innovation Center for Cancer Medicine, Guangdong Key Laboratory of Nasopharyngeal Carcinoma Diagnosis and Therapy, Sun Yat-sen University Cancer Center, Guangzhou, China; ^5^ Imaging Diagnosis and Interventional Center, Sun Yat-sen University Cancer Center, Guangzhou, China

**Keywords:** decrease rate, Epstein-Barr virus, nasopharyngeal carcinoma, induction chemotherapy, cumulative platinum dose

## Abstract

**Background/Objective:**

We aimed to explore the prognostic value of regression rate (RR) of plasma Epstein–Barr virus (EBV) DNA after induction chemotherapy (IC) in patients with stages II–IVA nasopharyngeal carcinoma (NPC).

**Methods:**

Eligible patients receiving IC followed by concurrent chemoradiotherapy were included. The cut-off value of pre-treatment EBV DNA (pre-IC DNA) and RR were identified by receiver operating curve (ROC). Recursive partitioning analysis (RPA) was applied to create new staging. Harrell’s c-index and time-independent ROC were employed to compare different RPA staging.

**Results:**

In total, 1,184 patients were included. The cut-off values of pre-IC DNA and RR were 16,200 copies/ml and 95.127% for patients receiving two cycles, and 5,520 copies/ml and 99.994% for those receiving three cycles. Notably, we only focused on patients receiving two cycles of IC. Patients with a RR >95.127% had significantly better 5-year overall survival (OS) than those with a RR ≤95.127% (86.2% *vs*. 54.3%, *P <*0.001). Then, RPA1 (pre-IC DNA + TNM staging + RR) and RPA2 (pre-IC DNA + TNM staging + post-IC EBV DNA [post-IC DNA]) staging systems were created. RPA1 staging achieved stronger power in OS prediction than RPA2 staging and TNM staging (c-index: 0.763 [0.714–0.812] *vs*. 0.735 [0.684–0.786] *vs*. 0.677 [0.604–0.749]; AUC: 0.736 *vs*. 0.714 *vs*. 0.628), indicating that RR had stronger prognostic power than post-IC DNA. Moreover, patients with stages III–IV_RPA1_ could benefit from high concurrent cumulative platinum dose (≥160 mg/m^2^).

**Conclusion:**

RR in conjunction with current TNM staging could better conduct risk stratification, prognosis prediction and help to guide precise concurrent chemotherapy.

## Background

Recent epidemiological data on nasopharyngeal carcinoma (NPC) shows that its incidence in China accounts for nearly 50% of new cases worldwide (1). More than 70% of newly diagnosed cases present with locoregionally advanced disease (2), and induction chemotherapy (IC) followed by concurrent chemoradiotherapy (CCRT) has been established as the preferable treatment for this subpopulation based on recent clinical trials (3–7). However, long-term follow-up results from these trials showed that nearly 30% of patients would eventually experience treatment failure (5, 8). Therefore, it’s still important to investigate powerful prognostic factors which could help to identify high-risk patients and make precisely post-IC or post-CCRT treatments selection.

During the past two decades, plasma Epstein–Barr virus (EBV) DNA has been proven to be a robust prognostic factor in NPC (9–13). The wide application of plasma EBV DNA in clinical practice has made NPC a more controllable and chronic malignancy. Recent studies showed that the response of plasma EBV DNA to IC could predict the prognosis of patients receiving IC (i.e., patients with detectable plasma EBV DNA after IC [post-IC DNA] achieved significantly worse survival outcomes than those with undetectable post-IC DNA) (14, 15). These findings provided a feasible and effective way to conduct risk stratification during the course of treatment, therefore allowing the timely adjustment of subsequent treatments. However, do all patients with detectable post-IC DNA have poor outcomes? Clinical practice showed that a proportion of patients with high pre-treatment plasma EBV DNA (pre-IC DNA) but very low detectable post-IC DNA also achieved satisfactory outcomes. Therefore, it may not be ideal to employ detectable/undetectable as the threshold for post-IC DNA in performing prognosis prediction and risk stratification without considering pre-IC DNA.

Previous study pointed out that the clearance rate of plasma EBV DNA was correlated with tumor response and survival for metastatic/recurrent NPC patients (16). Here, we explored and validated regression rate (RR), which is defined as the change rate of plasma EBV DNA load after IC, as the threshold for post-IC EBV DNA and conduct this study to explore and validate the prognostic value of RR. Moreover, our study will also compare the prognostic ability of RR with previously reported threshold (detectable/undetectable) for post-IC DNA.

## Materials and Methods

### Study Patient

All patients recruited for this study were treated between 2009 and 2015 at our center and data were retrospectively collected. Patients meeting the following criteria were included: (i) newly diagnosed stages II–IVA disease (8th edition TNM staging system); (ii) treated by IC plus CCRT; (iii) IC cycle should be two or three; (iv) the concurrent chemotherapy regimen should be tri-weekly or weekly cisplatin/nedaplatin; (v) data on pre-IC DNA and post-IC DNA were available; (vi) receiving radical radiotherapy (≥66 Gy); (vii) adjuvant chemotherapy was not allowed. This study was approved by the Research Ethics Committee of Sun Yat-sen University Cancer Center and informed consent was obtained from all patients before treatment.

### Study Data Deposit

The authenticity of this article has been validated by uploading the key raw data onto the Research Data Deposit (RDD) public platform (www.researchdata.org.cn), with the approval RDD number as RDDA2020001461.

### Staging Workup

Pre-treatment evaluation for tumor staging at our center included imaging methods, plasma EBV DNA load and physical examination of head and neck. Imaging methods included nasopharyngoscopy, magnetic resonance imaging (MRI) of head and neck, chest radiography or computed tomography (CT), abdominal ultrasound or CT, whole-body bone scan or positron emission tomography (PET)-CT. Imaging information were reviewed by two radiologists (Y-XM and X-HW) at our center, and all patients were staged according to the 8th edition of the International Union against Cancer/American Joint Committee on Cancer (UICC/AJCC) staging system (17).

### Plasma EBV DNA Quantification

Real-time quantitative polymerase chain reaction (qPCR) was used to quantify plasma EBV DNA load and the procedure was performed as previously described (18). Briefly, peripheral blood samples (about 3 ml) before IC and 7–21 days after IC were collected from each patient. Total DNA was extracted from 500 ul blood sample and the *BamH* I-W region of EBV genome was targeted for qPCR assay. The RR was calculated using the following equation: RR = (C_pre-IC DNA_ − C_post-IC DNA_)/C_pre-IC DNA_ × 100%, in which C_pre-IC DNA_ represented the load before IC while C_post-IC DNA_ represented the load after IC. Of note, RR was not calculated for patients with undetectable pre-IC DNA.

### Induction and Concurrent Chemotherapy

IC was delivered every 3 weeks for two to three cycles. The IC regimens were platinum-based chemotherapy consisting of gemcitabine plus cisplatin/nedaplatin (GP), docetaxel plus cisplatin/nedaplatin (TP), 5-fluorouracil plus cisplatin/nedaplatin (PF) or triple combination of docetaxel plus 5-fluorouracil with cisplatin/nedaplatin (TPF). Notably, IC may also be delivered to patients with stage II disease in our center if they have pre-treatment EBV DNA load >4,000 copies/ml or large metastatic lymph node sizes (>3 cm but <6 cm), because patients with these two factors have high risk of developing distant metastasis. Concurrent chemotherapy was single-agent cisplatin/nedaplatin delivered during radiotherapy every three weeks (80–100 mg/m^2^) for one to three cycles or every week (20–40 mg/m^2^) for one to seven cycles.

### Radiotherapy

All patients received intensity-modulated radiotherapy (IMRT) delivered by simultaneous integrated boost (SIB). Prescribed radiation dose was 68–70 Gy/30–33 f to the planning tumor volume (PTV) of primary tumor, 64–70 Gy/30–33 f to the PTV of metastatic lymph node, 60–63 Gy/28–33 f to the PTV of high-risk clinical target volume and 50–56 Gy/28–33 f to the PTV of low-risk clinical target volume.

### Follow-Up and Study Endpoints

According to the guidelines of our center, patients were followed every 3–6 months during the first 2 years, then 6–12 months during the third to fifth year and every year thereafter. Follow-up workups included physical examination, imaging methods (MRI of head and neck, chest radiography or CT, abdominal ultrasound or CT, whole-body bone scan) and plasma EBV DNA. Local or regional recurrence was diagnosed by pathology whenever possible, while distant metastasis was mainly confirmed by imaging findings if pathology was unavailable.

Endpoints evaluated in our study included overall survival (OS, time interval between diagnosis and death from any cause), disease-free survival (DFS, defined as time interval between diagnosis and disease progression including non-cancer death), distant failure-free survival (DFFS, time interval between diagnosis and distant failure) and locoregional failure-free survival (LRFFS, time interval between diagnosis and local or regional failure or both).

### Statistical Method

Difference of categorical and continuous variables between groups were compared using Chi-square or Fisher’s exact test and non-parametric test, respectively. To identify the threshold values for pre-IC DNA and RR that were most closely associated with OS, we employed receiver operating curve (ROC) analysis. The values with the biggest AUCs (sensitivity plus specificity) were selected as the cut-off values for model construction. Survival outcomes were established by Kaplan–Meier method and differences were compared by Log-rank test. Independent prognostic factors and corresponding hazard ratios (HRs) and 95% confidence intervals (CIs) were identified by Cox proportional hazard model. Patients were subdivided into different risk groups based on pre-IC DNA and RR. Recursive partitioning analysis (RPA) for OS was applied to create new staging systems on the basis of TNM staging, pre-IC DNA and post-IC DNA or RR. To compare the prognostic ability of RR with post-IC DNA (detectable/undetectable), we would build two RPA staging systems using these factors. Harrell’s C-index and time-independent ROC were employed to compare their prognostic ability. To avoid overfitting, we assessed internal validity with a bootstrapping procedure to calculate the relatively adjusted C-index and 5-year ROC for estimate of the performance of these factors in similar future patients. In the bootstrapping validation, the random resampling size was equal to the sample size of the original set and duplicates were allowed. To obtain a more comprehensive measure, we conducted the above procedure 10 times and presented the average value as well as the range of the results. All tests were two-sided and *P <*0.05 was considered significant. R software (version 3.6.3, http://www.Rproject.org) and Stata Statistical Package 12 (StataCorp LP, College Station, TX, USA) were used for all analyses.

## Results

### Patient Information

In total, 1,184 patients treated between October 2009 and December 2015 were included in this study (**Supplementary Figure S1**). The baseline information of these patients are presented in **Supplementary Table S1**. There were 878 (74.2%) male patients, and the median age for the whole cohort was 43 (range, 7–76) years. Approximately half of the patients were stage IVA (597, 50.4%) with the remaining patients divided between Stages III (496, 41.9%) and II (91, 7.7%). For IC regimen, most patients were treated with TPF (716, 60.5%) while the remainder were split between TP (115, 9.7%), PF (296, 25.0%), and GP (57, 4.8%). Moreover, 625 (52.8%) patients received two cycles and 559 (47.2%) patients received three cycles of IC. Median cumulative platinum dose (CPD) was 160 mg/m^2^ (range, 20–300 mg/m^2^).

Median follow-up duration of the cohort was 62.5 (range, 4.3–121.8) months. In total, 231 (19.5%) patients developed distant failure, 108 (9.1%) experienced local recurrence, 90 (7.6%) suffered regional recurrence and 277 (23.4%) patients died. An estimated 5-year OS, DFS, DFFS and LRFFS rates were 80.7, 72.2, 81.0 and 86.3%, respectively (**Supplementary Figure S2**).

### Pre-IC DNA, Post-IC DNA and RR

In our study, the plasma EBV DNA levels after the 2nd cycle of IC were used for analysis for patients receiving two cycles of IC while the corresponding levels after 3rd cycle of IC were used for those receiving three cycles. Among the 1,184 patients, 201 (17.0%) had undetectable pre-IC DNA and 859 (72.6%) had undetectable post-IC DNA (baseline information between undetectable and detectable post-IC patients were shown in **Supplementary Table S2**). Median pre-IC DNA and post-IC DNA load were 6,870 copies/ml (range, 0–19,500,000 copies/ml) and 0 copy/ml (range, 0–61,600,000 copies/ml), respectively. Of the 859 patients with detectable pre-IC DNA, 675 (68.9%) had undetectable post-IC DNA. A small portion (61, 5.2%) of patients experienced increases in their post-IC DNA levels compared to pre-IC DNA levels which were undetectable in 17 (1.4%) and detectable in 44 (3.7%) patients, respectively.

**Figure 1:**
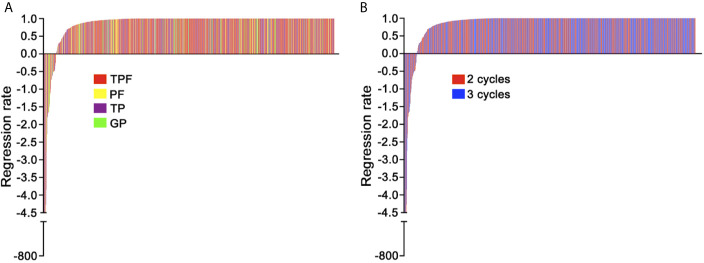
**(A)** Waterfall plot of decrease rate for each patient between different induction chemotherapy regimens; TPF, docetaxel plus cisplatin/nedaplatin and 5-fluorouracil; PF, cisplatin/nedaplatin plus 5-fluorouracil; TP, docetaxel plus cisplatin/nedaplatin; GP, gemcitabine plus cisplatin; **(B)** Waterfall plot of decrease rate for each patient between different cycles.

Of the 983 patients with detectable pre-IC DNA levels, the interquartile range of RR was 97.5 to 100% and median RR was complete response (100% decline) for all regimens (TPF [interquartile range, 99.4 to 100%], PF [interquartile range, 96.8 to 100%], TP [interquartile range, 90.8 to 100%], GP [interquartile range, 97.8 to 100%]). RR was comparable for all regimens with no regimen significantly better or worse (all *P >*0.05, **Supplementary Table S3** and **Figure 1A**). However, duration of chemotherapy was marginally significant with patients receiving two vs three cycles of IC having slightly different rates of decline (*P* = 0.052; **Figure 1B**).

### Cut-Off Value of Pre-IC DNA, Post-IC DNA and RR

**Figure 2:**
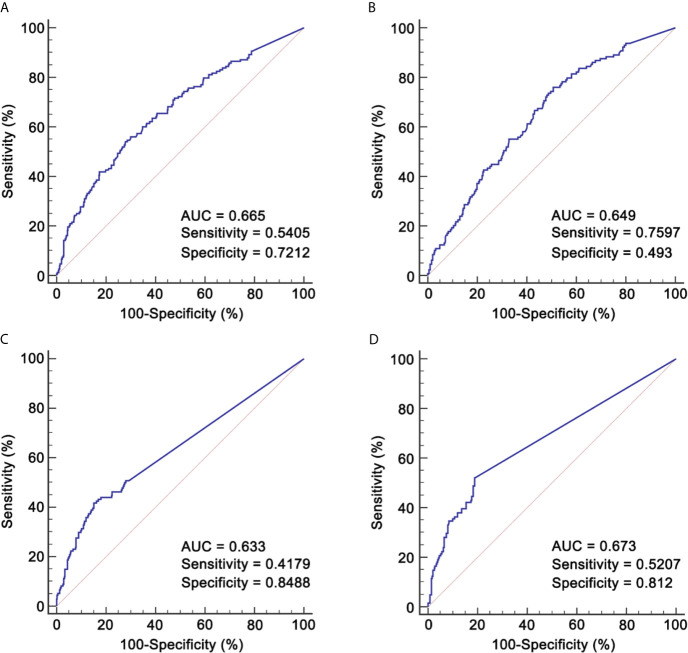
ROCs for identifying cut-off values of pre-IC DNA and RR. **(A)** Pre-IC DNA in patients receiving 2 cycles of IC; **(B)** Pre-IC DNA in patients receiving 3 cycles of IC; **(C)** RR in patients receiving 2 cycles of IC; **(D)** RR in patients receiving 3 cycles of IC. ROC, receiver operating curve; pre-IC DNA, pre-treatment Epstein-Barr virus DNA; RR, decrease rate; IC, induction chemotherapy; AUC, area under curve.

Given the potential effect of IC cycle on RR, we explored the role of RR in patients receiving two and three cycles separately. The cut-off value of pre-IC DNA was 16,200 copies/ml (area under curve [AUC] = 0.665; **Figure 2A**) for patients receiving two cycles and 5,520 copies/ml (AUC = 0.649; **Figure 2B**) for those receiving three cycles. Moreover, the corresponding cut-off values of RR was 95.127% (AUC = 0.633; **Figure 2C**) and 99.994% (AUC = 0.673; **Figure 2D**). For post-IC DNA, 0 copy/ml would be used as the cut-off value. Univariate survival analysis showed that all the four cut-off values had strong power in stratifying patients into different risk groups which achieved significantly different OS (**Supplementary Figure S3**). Moreover, patients with detectable post-IC DNA had significantly worse OS, DFS, DFFS and LRFFS than those with undetectable post-IC DNA (**Supplementary Figure S4**). For those receiving two cycles of IC, patients with a RR >95.127% had significantly better a 5-year OS rate than those with a RR ≤95.127% (86.2% *vs*. 54.3%, *P <*0.001). Because the cut-off value of RR in patients receiving three cycles of IC was too close to 100% (namely undetectable) and only one patient had a RR between 99.994 and 100% (the other were 100%), we would not discuss the role of RR in this subpopulation.

### Prognostic Value of RR in Patients Receiving Two Cycles of IC

Based on pre-IC DNA and RR, patients were stratified into eight groups (**Supplementary Table S4**) and their baseline information are presented in **Supplementary Table S5**. Univariate survival outcomes of these eight groups are shown in **Supplementary Figure S5** and **Supplementary Table S6**. As shown, survival outcomes were not significantly different between groups 3 and 4 and between groups 6 and 7, indicating that patients with a RR between 95.127 and 100% also could achieve comparable outcomes as those with a RR = 100%. As expected, groups 1, 3 and 4 had significantly better survival outcomes than the other groups, and group 8 achieved the worst outcomes with the 5-year OS of 39.1%. Multivariate analysis also found similar results (**Supplementary Table S7**).

**Figure 3:**
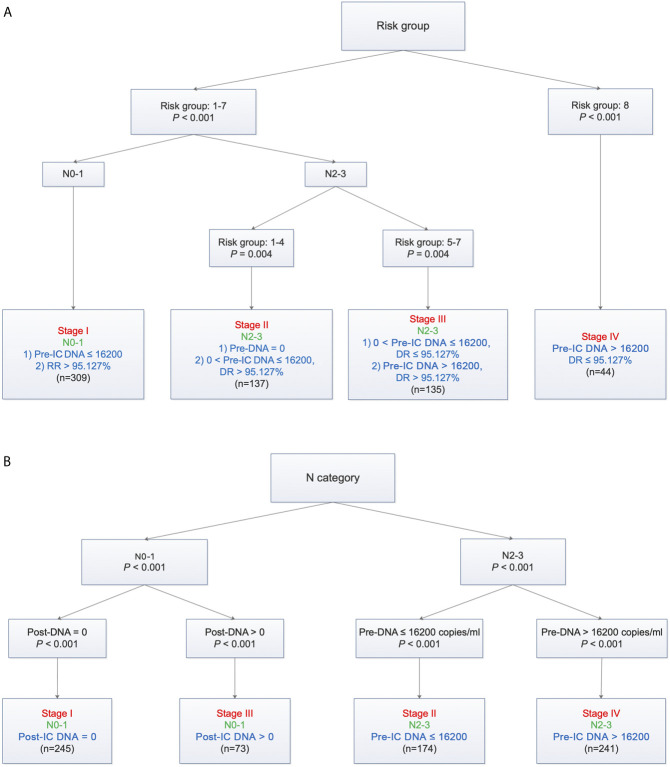
Recursive partitioning analysis to create new staging system after combining decrease rate **(A)** or post-IC DNA **(B)** with pre-IC DNA and TNM staging. **(A)** RPA1 staging system; **(B)** RPA2 staging system. Pre-IC DNA, pre-treatment Epstein-Barr virus DNA; RR, decrease rate; Post-IC DNA, plasma Epstein-Barr virus DNA after induction chemotherapy.

**Figure 4:**
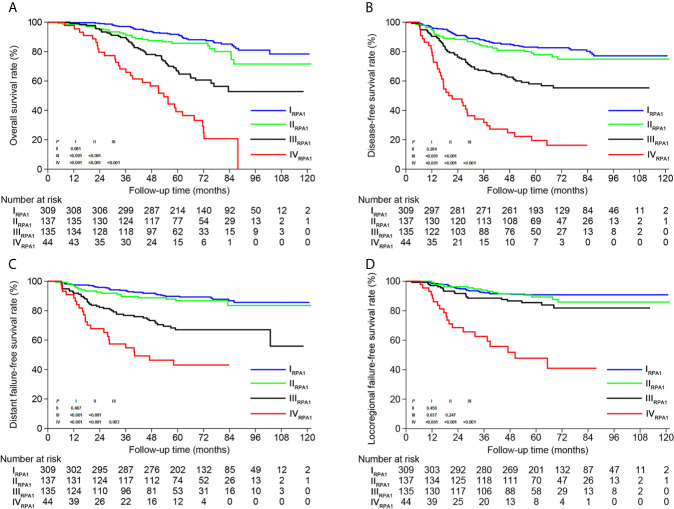
Kaplan-Meier survival curves of **(A)** overall survival, **(B)** disease-free survival, **(C)** distant failure-free survival and **(D)** locoregional failure-free survival between different risk groups of RPA1 staging.

Based on TNM staging and the risk groups, we created one RPA staging system which consisted of four groups (RPA1, **Table 1** and **Figure 3A**). We also built another RPA staging system on the basis of TNM staging, post-IC DNA (detectable vs. undetectable) and pre-IC DNA (RPA2, **Table 1** and **Figure 3B**). The 5-year OS rates were 91.8, 85.7, 66.9 and 39.1% for stages I, II, III, and IV of the RPA1 staging system respectively (**Figure 4** and **Supplementary Table S8** **)**; and were 93.8, 79.8%, 79.8, and 60.8% for stages I, II, III, and IV of the RPA2 staging system (**Supplementary Figure S6** and **Supplemetary Table S8**).

**Table 1:** Definition of different RPA staging.

Staging	Definition	Classification	5-year OS
RPA1	1) N0-1, Pre-IC DNA ≤ 16200 copies/ml	I	91.8%
	2) N0-1, RR > 95.127%		
	1) N2-3, Pre-IC DNA = 0	II	85.7%
	2) N2-3, 0 < Pre-IC DNA ≤ 16200 copies/ml, RR > 95.127%		
	1) N2-3, 0 < Pre-IC DNA ≤ 16200 copies/ml, RR ≤ 95.127%	III	66.9%
	2) N2-3, Pre-IC DNA > 16200 copies/ml, RR > 95.127%		
	Pre-IC DNA > 16200 copies/ml, RR ≤ 95.127%	IV	39.1%
RPA2	N0-1, Post-IC DNA = 0	I	93.8%
	N2-3, Pre-IC DNA ≤ 16200 copies/ml	II	79.8%
	N0-1, Post-IC DNA > 0	III	79.8%
	N2-3, Pre-IC DNA > 16200 copies/ml	IV	60.8%
	T1N1/T2N0-1	II	91.2%
TNM	T3N0-2/T1-2N2	III	87.2%
	T4N0-3/T1-3N3	IV	72.8%

Pre-IC DNA, pre-treatment Epstein-Barr virus DNA load; Post-IC DNA, plasma Epstein-Barr virus DNA load after induction chemotherapy; RR, regression rate; OS, overall survival.

**Figure 5:**
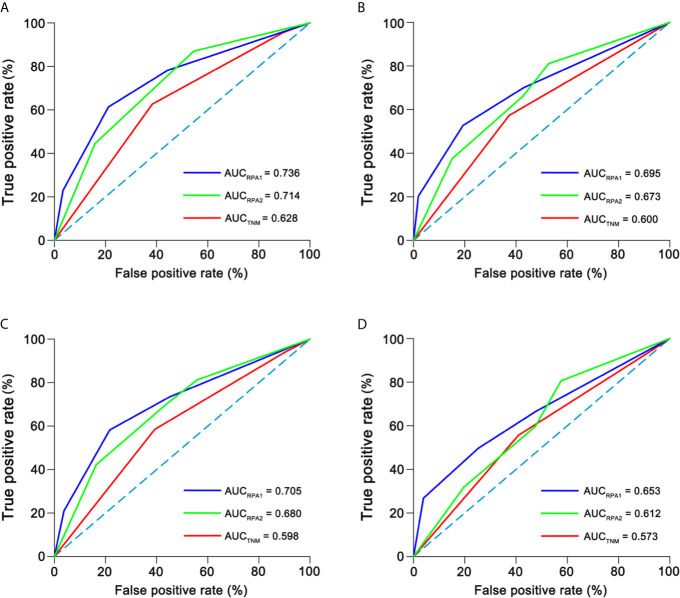
Time-independent ROCs of different staging. **(A)** 5-year overall survival; **(B)** 5-year disease-free survival; **(C)** 5-year distant failure-free survival; **(D)** 5-year locoregional failure-free survival. ROC, receiver operating curve; AUC, area under curve.

The c-indexes of 5-year OS and DFS were 0.763 (0.714–0.812) and 0.731 (0.684–0.778) for RPA1 staging, 0.735 (0.684–0.786) and 0.698 (0.650–0.747) for RPA2 staging and 0.677 (0.604–0.749) and 0.631 (0.565–0.696) for TNM staging (**Supplementary Table S9**). With regard to time-independent ROC analysis, the AUCs of 5-year OS ROCs were 0.736 for RPA1 staging, 0.714 for RPA2 staging and 0.628 for TNM staging (**Figure 5**). Also, RPA1 staging achieved the highest AUCs for the other survival endpoints (**Figure 5**). Obviously, RPA1 staging achieved the best prognostic discrimination power, indicating that RR outperformed post-IC DNA (detectable vs. undetectable) and further supporting the notion that RR could serve as a powerful prognostic factor and a supplement to TNM staging.

To avoid overfitting of our model, we performed random bootstrap analysis to validate the thresholds for pre-IC DNA and RR, and then validate our model. We first performed bootstrap analysis 100 times and the corresponding thresholds were shown in **Supplementary Table S10**. The average threshold of Pre-IC DNA was 16,060 copies/ml which was very close to our previously identified threshold which was calculated based the whole cohort (16,200 copies/ml). With regard to RR, its threshold had a 37% repeatability and the average threshold was 95.163% which is also very close to our previous threshold (95.127%). These results suggested that our models built on these thresholds were valid and reproducible.

Next, we presented the c-indexes and AUCs of the first ten analyses (**Supplementary Tables S11**, **S12**) and only showed one of them here. The c-indexes of 5-year OS and DFS were 0.771 (0.721–0.822) and 0.744 (0.697–0.791) for RPA1 staging, 0.749 (0.696–0.801) and 0.724 (0.675–0.773) for RPA2 staging, and 0.674 (0.600–0.749) and 0.664 (0.600–0.729) for TNM staging (**Supplementary Table S12**). With regard to time-independent ROC analysis, the AUCs of 5-year OS ROCs were 0.74 for RPA1 staging, 0.712 for RPA2 staging and 0.611 for TNM staging (**Supplementary Table S11** and **Supplementary Figure S7**). RPA1 also achieved the highest AUCs for the other endpoints (**Supplementary Figure S7**). These results further suggested that RPA1 staging had the strongest power.

**Figure 6:**
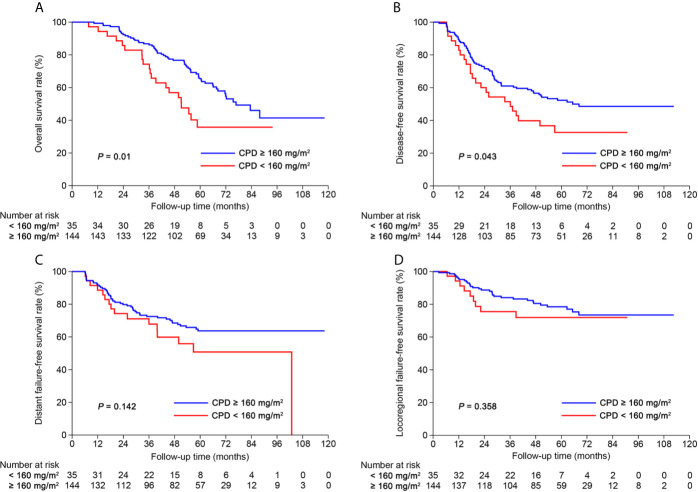
Kaplan-Meier curves of **(A)** overall survival, **(B)** disease-free survival, **(C)** distant failure-free survival and **(D)** locoregional failure-free survival for patients with stage III-IV_RPA1_ receiving cumulative platinum dose < or ≥ 160 mg/m^2^.

After risk stratification, we further evaluated whether the RPA staging could predict the benefit of cumulative platinum dose (CPD). When stratified by TNM staging, patients could not benefit from high CPD (≥160 mg/m^2^; **Supplementary Figure S8**). When stratified by RPA1 staging, patients with stages III–IV_RPA1_ could obtain OS (5-year: 65.6% *vs*. 35.8%, *P* = 0.01) and DFS (5-year: 52.3% *vs*. 32.7%, *P* = 0.043; **Figure 6**) benefits from high CPD while patients with stages I–II_RPA1_ could not (**Supplementary Figure S9**). Results of multivariate analysis also confirmed these findings (**Supplementary Tables S13**, **S14**).

## Discussion

To the best of our knowledge, our current study is the first one to explore the prognostic value of RR of post-IC plasma EBV DNA in patients with stages II–IVA NPC receiving two cycles of IC and found that patients with a RR >95.127% achieved significantly better survival outcomes than those with a RR ≤95.127%. Based on pre-IC DNA, RR and TNM staging, we created a new RPA staging system which stratified patients into four risk groups and outperformed TNM staging. By comparing RPA1 staging with RPA2 staging, we demonstrated that RR had stronger prognostic power than post-IC DNA (detectable vs. undetectable). Moreover, the RPA1 staging could predict the benefit of CPD (≥ *vs*. <160 mg/m^2^).

We should pointed out that it may be inappropriate to compare our RPA staging with TNM staging because the former included prognostic factors both before and post treatment while the latter only included pre-treatment factors. However, this should not affect our conclusions since the main purpose of our study is to determine the prognostic value of RR and evaluate whether it could serve as a complement to the TNM staging to improve its power in prognosis prediction. Our results truly revealed that the new RPA staging after integration of RR has stronger power than the TNM staging alone. These results indicated that RR could serve as a complement to TNM staging to predict post-treatment prognosis.

The monitoring of plasma EBV DNA before, during and after treatment has improved prognostication and enabled careful treatment selection such as personalized IC (19, 20). Recent reports on plasma EBV DNA showed that post-IC DNA could act as a strong prognostic biomarker for patients receiving IC plus CCRT (14, 15, 21, 22). These findings provided new insights into the supervision of treatment efficacy and risk stratification during treatment, therefore allowing timely modifications of subsequent treatments. However, previous threshold used for post-IC DNA is usually detectable/undetectable (13–15) which may be insufficient to identify whether patients were sensitive to treatment. For example, patients experiencing dramatic decrease in plasma EBV DNA after IC (i.e., very high pre-IC DNA but very low post-IC DNA) may also be sensitive to treatment although they did not have undetectable post-IC DNA. Actually, this has been proved by our results that patients in group 7 (high pre-IC DNA but low post-IC DNA) had comparable survival outcomes to those in group 6 (high pre-IC DNA and undetectable post-IC DNA). Moreover, we further proved that RR had stronger power than post-IC DNA (detectable vs. undetectable) in risk stratification by establishing and comparing two RPA staging systems (c-index: 0.763 *vs*. 0.735; 5-year OS AUC: 0.736 *vs*. 0.714). Together, these findings suggest that RR may be a better indicator to identify those who were sensitive or insensitive to treatments.

Our results showed that patients receiving three cycles of IC had marginally higher RR than those receiving two cycles (*P* = 0.052). To avoid potential impact of IC cycle on our model, we therefore constructed models separately among patients receiving two and three cycles. The underlying biological explanation for the difference, if there is one, may be that some patients have relatively slow but sustained response to IC, meaning that some patients could still response to the third cycle of IC. However, recent evidences suggested that patients achieved complete biological response (undetectable plasma EBV DNA) after two cycles of IC could not benefit from three or more cycles of IC (21). Therefore, it may be better to perform treatment efficacy evaluation after two cycles to evaluate whether some patients still need the third cycle of IC. Possibly, patients with RR less than 95.127% after two cycles of IC may be suitable to receive three cycles of IC. One thing should be pointed out is that we did not evaluate the prognostic value of RR in patients receiving three cycles of IC because the cut-off value of 99.994% is too close to 100% which represents undetectable post-IC DNA. Moreover, only one patient in our study had a RR between 99.994 and 100%, meaning that the RPA staging system built on RR almost does not differ from that built on post-IC DNA. In light of this, it may be redundant to build a model using RR but not post-IC DNA for patients receiving three cycles of IC.

CPD has been proven as an important treatment consideration in NPC (23). In light of this, the purpose of investigating RR is not only to conduct post-IC risk stratification but also to guide accurate selection for concurrent chemotherapy. Therefore, our study identified the subpopulation who could benefit from high CPD (≥160 mg/m^2^) according to RPA1 staging. As shown, the TNM staging failed to predict the benefit of high CPD. When stratified by RPA1 staging, patients with stage III-IV_RPA1_ could benefit from high CPD while those with stages I–II could not. This evidence further supports that RR may be an ideal biomarker for risk stratification and precise treatment selection.

One main strength of our study is that all patients were treated by IC plus CCRT and the radiotherapy was delivered using IMRT, which could reduce treatment-related bias. Limitations should also be acknowledged. First, the retrospective nature may subject our study to potential bias. Second, IC regimens were different. However, this should not affect our results because OS and RRs were comparable between the four IC regimens. Third, nedaplatin was used concurrently with radiotherapy. One recent clinical trial showed that nedaplatin had similar efficacy but less toxicities compared with cisplatin (24). Therefore, the usage of nedaplatin should have no or very limited impact on our findings. Finally, large sample size may be needed to further address the role of RR in patients receiving three cycles of IC.

## Conclusion

Overall, our current study explored and validated the role of RR for patients with stages II–IVA NPC receiving two cycles of IC in the era of IMRT. The established RPA1 staging based on RR, pre-IC DNA and TNM staging could help to identify risk stratification and guide precise concurrent chemotherapy.

## Data Availability Statement

The datasets presented in this study can be found in online repositories. The names of the repository/repositories and accession number(s) can be found below: https://www.researchdata.org.cn/Search.aspx?Num=RDDA2020001461, RDDA2020001461.

## Ethics Statement

The studies involving human participants were reviewed and approved by Sun Yat-sen University Cancer Center. Written informed consent for participation was not required for this study in accordance with the national legislation and the institutional requirements.

## Author Contributions

HP and FH contributed to study design. HP, B-bC and Y-XM collected the study data. HP, B-bC, and X-hW contributed to data analysis and interpretation. HP and B-bC contributed to manuscript writing. X-hW and FH reviewed the manuscript and contributed to quality control. All authors contributed to the article and approved the submitted version.

## Funding

This work was funded by the National Natural Science Foundation of China (82002981).

## Conflict of Interest

The authors declare that the research was conducted in the absence of any commercial or financial relationships that could be construed as a potential conflict of interest.

## Supplementary Material

Supplementary Table 1 Baseline information of 1184 patients with nasopharyngeal carcinoma receiving induction chemotherapy plus concurrent chemoradiotherapy.

Supplementary Table 2 Baseline characteristics between patients with detectable and undetectable post-IC EBV DNA.

Supplementary Table 3 Comparison of decrease rate between different induction chemotherapy regimens.

Supplementary Table 4 Risk stratification based on pre-IC DNA and decrease rate for patients receiving 2 cycles of IC.

Supplementary Table 5 Baseline information of the 8 groups.

Supplementary Table 6 Estimated 5-year survival outcomes of the 8 groups.

Supplementary Table 7 Results of multivariate survival analysis.

Supplementary Table 8 Estimated 5-year survival outcomes of different RPA staging.

Supplementary Table 9 C-index of different staging systems.

Supplementary Table 10 AUC of all bootstrap analysis.

Supplementary Table 11 C-indexes of all bootstrap analysis.

Supplementary Table 12 Results of multivariate analysis among patients with stage III-IV_RPA1_.

Supplementary Table 13 Results of multivariate analysis among patients with stage I-II_RPA1_.

Supplementary Figure 1 Flow diagram of patient inclusion. NPC, nasopharyngeal carcinoma; IMRT, intensity-modulated radiotherapy; IC, induction chemotherapy; CCRT, concurrent chemoradiotherapy; pre-IC DNA, pre-treatment Epstein-Barr virus DNA; post-IC DNA, plasma Epstein-Barr virus DNA after induction chemotherapy. ^#^The prescribed radiation dose of the gross tumor volume was ≥ 66Gy. *Patients were staged according to the 8th edition of the International Union against Cancer/American Joint Committee on Cancer (UICC/AJCC) staging system.

Supplementary Figure 2 Kaplan-Meier survival curves of **(A)** overall survival, **(B)** disease-free survival, **(C)** distant failure-free survival and **(D)** locoregional failure-free survival for the whole cohort.

Supplementary Figure 3 Overall survival curves between **(A)** pre-IC DNA > and ≤ 16200 copies/ml in patients receiving 2 cycles, **(B)** pre-IC DNA > and ≤ 5520 copies/ml in patients receiving 3 cycles, **(C)** RR > and ≤ 95.127% in patients receiving 2 cycles and **(D)** RR > and ≤ 95.127% in patients receiving 3 cycles. Pre-IC DNA, pre-treatment Epstein-Barr virus DNA; RR, regression rate.

Supplementary Figure 4 Kaplan-Meier survival curves of **(A)** overall survival, **(B)** disease-free survival, **(C)** distant failure-free survival and **(D)** locoregional failure-free survival between patients with detectable and undetectable post-IC DNA. Post-IC DNA, post-induction chemotherapy EBV DNA.

Supplementary Figure 5 Kaplan-Meier survival curves of **(A)** overall survival, **(B)** disease-free survival, **(C)** distant failure-free survival and **(D)** locoregional failure-free survival for the eight groups.

Supplementary Figure 6 Kaplan-Meier survival curves of **(A)** overall survival, **(B)** disease-free survival, **(C)** distant failure-free survival and **(D)** locoregional failure-free survival between different risk groups of RPA2 staging.

Supplementary Figure 7 Time-independent OS, DFS, DFFS and LRRFS ROCs of different staging in boostrap analysis. ROC, receiver operating curve; AUC, area under curve; OS, overall survival; DFS, disease-free survival; DFFS, distant failure-free survival; LRRFS, locoregional relapse-free survival.

Supplementary Figure 8 Kaplan-Meier curves of overall survival between patients with a cumulative platinum dose < or ≥ 160 mg/m^2^ within different stages. **(A)** Stage II; **(B)** Stage III; **(C)** Stage IVA; **(D)** Stage II/III; (E) Stage II/IVA; (F) Stage III/IVA.

Supplementary Figure 9 Kaplan-Meier curves of **(A)** overall survival, **(B)** disease-free survival, **(C)** distant failure-free survival and **(D)** locoregional failure-free survival between patients with stage I-II_RPA1_ receiving a cumulative platinum dose < or ≥ 160 mg/m^2^.
