# Supplementary material for: Prognostic Value of Regression Rate of Plasma EBV DNA After Induction Chemotherapy for Stage II-IVA Nasopharyngeal Carcinoma
Source: Front Oncol. 2021 Jul 15;11:689593. doi: 10.3389/fonc.2021.689593 (PMC8319726; doi:10.3389/fonc.2021.689593)
Supplement: Supplementary Table 1 — Baseline information of 1184 patients with nasopharyngeal carcinoma receiving induction chemotherapy plus concurrent chemoradiotherapy. [file DataSheet_2.docx]

**Supplementary Tables**

**Supplementary Table S1**. Baseline information of 1184 patients with nasopharyngeal carcinoma receiving induction chemotherapy plus concurrent chemoradiotherapy.

| Characteristics | No. (%) |
| --- | --- |
| Gender |  |
| Male | 878 (74.2) |
| Female | 306 (25.8) |
| Age |  |
| Median (range, y) | 43 (7-76) |
| Smoking |  |
| Yes | 429 (36.2) |
| No | 755 (63.8) |
| Drinking |  |
| Yes | 164 (13.9) |
| No | 1020 (86.1) |
| T category |  |
| T1 | 96 (8.1) |
| T2 | 139 (11.7) |
| T3 | 535 (45.2) |
| T4 | 414 (35.0) |
| N category |  |
| N0 | 59 (5.0) |
| N1 | 510 (43.1) |
| N2 | 363 (30.6) |
| N3 | 252 (21.3) |
| Overall stage |  |
| II | 91 (7.7) |
| III | 496 (41.9) |
| IVA | 597 (50.4) |
| Pre-IC DNA |  |
| Median (range, copies/ml) | 6870 (0-19500000) |
| ≥ 1000 (copies/ml) | 433 (69.3%) |
| < 1000 (copies/ml) | 192 (30.7%) |
| Post-IC DNA |  |
| Median (range, copies/ml) | 0 (0-61600000) |
| IC regimen |  |
| TPF | 716 (60.5) |
| TP | 115 (9.7) |
| PF | 296 (25.0) |
| GP | 57 (4.8) |
| IC cycle |  |
| 2 cycles | 625 (52.8) |
| 3 cycles | 559 (47.2) |
| Concurrent chemotherapy |  |
| Median cumulative platinum dose (mg/m^2^) | 160 (20-300) |

Abbreviations: Pre-IC DNA, plasma EBV DNA load before induction chemotherapy; Post-IC DNA, plasma EBV DNA load after induction chemotherapy; TPF, docetaxel plus cisplatin/nedaplatin and 5-fluorouracil; TP, docetaxel plus cisplatin/nedaplatin; PF, cisplatin/nedaplatin plus 5-fluorouracil; GP, gemcitabine plus cisplatin/nedaplatin.

**Supplementary Table S2.** Baseline characteristics between patients with detectable and undetectable post-IC EBV DNA.

| Characteristics | Detectable  (n=325, %) | Undetectable  (n=859, %) | *P* value |
| --- | --- | --- | --- |
| Gender |  |  | 0.655 |
| Male | 238 (73.2) | 640 (74.5) |  |
| Female | 87 (26.8) | 219 (25.5) |  |
| Age |  |  |  |
| Median (range, y) | 45 (13-76) | 43 (7-75) | 0.412 |
| Smoking |  |  | 0.054 |
| Yes | 132 (40.6) | 297 (34.6) |  |
| No | 193 (59.4) | 562 (65.4) |  |
| Drinking |  |  | 0.188 |
| Yes | 52 (16) | 112 (13.0) |  |
| No | 273 (84) | 747 (87.0) |  |
| T category ^a^ |  |  | 0.016 |
| T1 | 20 (6.1) | 76 (8.9) |  |
| T2 | 49 (15.1) | 90 (10.5) |  |
| T3 | 131 (40.3) | 404 (47.0) |  |
| T4 | 125 (38.5) | 289 (33.6) |  |
| N category ^a^ |  |  | < 0.001 |
| N0 | 11 (3.4) | 48 (5.6) |  |
| N1 | 112 (34.5) | 398 (46.4) |  |
| N2 | 98 (30.1) | 265 (30.8) |  |
| N3 | 104 (32) | 148 (17.2) |  |
| Overall stage ^a^ |  |  | < 0.001 |
| II | 16 (4.9) | 75 (8.7) |  |
| III | 108 (33.2) | 388 (45.2) |  |
| IVA | 201 (61.9) | 396 (46.1) |  |
| Pre-IC DNA |  |  | 0.022 |
| Median (range, copies/ml) | 4590 (0-19500000) | 20000 (0-18000000) |  |
| IC regimen |  |  | 0.03 |
| TPF | 181 (55.7) | 535 (62.3) |  |
| TP | 42 (12.9) | 73 (8.5) |  |
| PF | 90 (27.7) | 206 (24.0) |  |
| GP | 12 (3.7) | 45 (5.2) |  |
| IC cycle |  |  | 0.016 |
| 2 cycles | 190 (58.5) | 435 (50.6) |  |
| 3 cycles | 135 (41.5) | 424 (49.4) |  |
| Concurrent chemotherapy |  |  | 0.486 |
| CCD (mg/m^2^) | 160 (20-300) | 160 (25-300) |  |

Abbreviations: IC, induction chemotherapy; TPF, docetaxel plus cisplatin and 5-fluorouracil; TP, docetaxel plus cisplatin; PF, cisplatin plus 5-fluorouracil; GP, gemcitabine plus cisplatin; CCD, cumulative cisplatin dose.

^a^ According to the 8^th^ edition of UICC/AJCC staging system.

**Supplementary Table S3.** Comparison of decrease rate between different induction chemotherapy regimens.

|  |  | TPF | PF | TP |
| --- | --- | --- | --- | --- |
|  |  | 100% (-2973.2% to 100%) | 100% (-668.4% to 100%) | 100% (-74206.4% to 100%) |
| PF | 100% (-668.4% to 100%) | *P* = 0.222 |  |  |
| TP | 100% (-74206.4% to 100%) | *P* = 0.458 | *P* = 0.227 |  |
| GP | 100% (-163.9% to 100%) | *P* = 0.851 | *P* = 0.085 | *P* = 0.384 |

Abbreviations: TPF, docetaxel plus cisplatin/nedaplatin with 5-fluorouracil; PF, cisplatin/nedaplatin plus 5-fluorouracil; TP, docetaxel plus cisplatin/nedaplatin; GP, gemcitabine plus cisplatin.

**Supplementary Table S4**. Risk stratification based on pre-IC DNA and decrease rate for patients receiving 2 cycles of IC.

| Pre-IC DNA (copies/ml) | Post-IC DNA/RR | Group | No. of patients |
| --- | --- | --- | --- |
| 0 | Post-IC DNA = 0 | 1 | 102 |
| 0 | Post-IC DNA > 0 | 2 | 12 |
| > 0, ≤ 16200 | Decrease rate = 100% | 3 | 213 |
| > 0, ≤ 16200 | 100% > Decrease rate > 95.127% | 4 | 18 |
| > 0, ≤ 16200 | Decrease rate ≤ 95.127% | 5 | 67 |
| > 16200 | Decrease rate = 100% | 6 | 120 |
| > 16200 | 100% > Decrease rate > 95.127% | 7 | 49 |
| > 16200 | Decrease rate ≤ 95.127% | 8 | 44 |

Abbreviations: Pre-IC DNA, pre-treatment Epstein-Barr virus DNA; Post-IC DNA, plasma Epstein-Barr virus DNA after induction chemotherapy; RR, regression rate; IC, induction chemotherapy.

**Supplementary Table S5.** Baseline information of the 8 groups.

| Characteristics | Group 1 | Group 2 | Group 3 | Group 4 | Group 5 | Group 6 | Group 7 | Group 8 |
| --- | --- | --- | --- | --- | --- | --- | --- | --- |
|  | (n=102) | (n=12) | (n=213) | (n=18) | (n=67) | (n=120) | (n=49) | (n=44) |
|  | No.(%) | No.(%) | No.(%) | No.(%) | No.(%) | No.(%) | No.(%) | No.(%) |
| Gender |  |  |  |  |  |  |  |  |
| Male | 75 (73.5) | 10 (83.3) | 157 (73.7) | 12 (66.7) | 45 (67.2) | 89 (74.2) | 38 (77.6) | 30 (68.2) |
| Female | 27 (26.5) | 2 (16.7) | 56 (26.3) | 6 (33.3) | 22 (32.8) | 31 (25.8) | 11 (22.4) | 14 (31.8) |
| Age |  |  |  |  |  |  |  |  |
| Median (range, y) | 43 (7-65) | 43 (26-58) | 42 (9-75) | 45 (19-64) | 47 (13-66) | 43 (15-66) | 45 (22-65) | 46 (20-68) |
| Smoking |  |  |  |  |  |  |  |  |
| Yes | 34 (33.3) | 4 (33.3) | 83 (39.0) | 9 (50.0) | 22 (32.8) | 44 (36.7) | 25 (51.0) | 17 (38.6) |
| No | 68 (66.7) | 8 (66.7) | 130 (61.0) | 9 (50.0) | 45 (67.2) | 76 (63.3) | 24 (49.0) | 27 (61.4) |
| Drinking |  |  |  |  |  |  |  |  |
| Yes | 14 (13.7) | 5 (41.7) | 25 (11.7) | 5 (27.8) | 8 (11.9) | 19 (15.8) | 8 (16.3) | 4 (9.1) |
| No | 88 (86.3) | 7 (58.3) | 188 (88.3) | 13 (72.2) | 59 (88.1) | 101 (84.2) | 41 (83.7) | 40 (90.9) |
| T category |  |  |  |  |  |  |  |  |
| T1 | 12 (11.8) | 1 (8.3) | 22 (10.3) | 1 (5.6) | 9 (13.4) | 11 (9.2) | 2 (4.1) | 1 (2.3) |
| T2 | 11 (10.8) | 0 (0) | 28 (13.1) | 4 (22.2) | 9 (13.4) | 11 (9.2) | 6 (12.2) | 11 (25.0) |
| T3 | 57 (55.9) | 6 (50.0) | 102 (47.9) | 9 (50.0) | 33 (49.3) | 63 (52.5) | 25 (51.0) | 15 (34.1) |
| T4 | 22 (21.5) | 5 (41.7) | 61 (28.7) | 4 (22.2) | 16 (23.9) | 35 (29.1) | 16 (32.7) | 17 (38.6) |
| N category |  |  |  |  |  |  |  |  |
| N0 | 13 (12.8) | 1 (8.3) | 10 (4.7) | 0 (0) | 3 (4.5) | 4 (3.3) | 0 (0) | 0 (0) |
| N1 | 56 (54.9) | 5 (41.7) | 114 (53.5) | 9 (50.0) | 27 (40.3) | 48 (40.0) | 19 (38.8) | 9 (20.4) |
| N2 | 24 (23.5) | 3 (25.0) | 65 (30.5) | 5 (27.8) | 17 (25.4) | 41 (34.2) | 16 (32.6) | 19 (43.2) |
| N3 | 9 (8.8) | 3 (25.0) | 24 (11.3) | 4 (22.2) | 20 (29.8) | 27 (22.5) | 14 (28.6) | 16 (36.4) |
| Overall stage |  |  |  |  |  |  |  |  |
| II | 16 (15.7) | 0 (0) | 24 (11.3) | 2 (11.2) | 6 (9.0) | 6 (5.0) | 1 (2.0) | 3 (6.8) |
| III | 57 (55.9) | 5 (41.7) | 115 (54.0) | 8 (44.4) | 27 (40.3) | 53 (44.2) | 22 (44.9) | 12 (27.3) |
| IVA | 29 (28.4) | 7 (58.3) | 74 (34.7) | 8 (44.4) | 34 (50.7) | 61 (50.8) | 26 (53.1) | 29 (65.9) |
| IC regimen |  |  |  |  |  |  |  |  |
| TPF | 49 (48.0) | 7 (58.3) | 112 (52.6) | 7 (38.9) | 35 (52.3) | 55 (45.8) | 22 (44.9) | 18 (40.9) |
| PF | 17 (16.7) | 0 (0) | 23 (10.8) | 3 (16.7) | 8 (11.9) | 18 (15.0) | 11 (22.4) | 12 (27.3) |
| TP | 36 (35.3) | 5 (41.7) | 77 (36.2) | 6 (33.3) | 24 (35.8) | 45 (37.5) | 15 (30.6) | 14 (31.8) |
| GP | 0 (0) | 0 (0) | 1 (0.4) | 2 (11.1) | 0 (0) | 2 (1.7) | 1 (2.1) | 0 (0) |
| Concurrent chemotherapy |  |  |  |  |  |  |  |  |
| Median CPD (mg/m^2^, range) | 160 (40-300) | 160 (120-240) | 160 (40-300) | 160 (75-240) | 160 (40-220) | 160 (20-300) | 180 (25-300) | 160 (60-300) |

Abbreviations: TPF, docetaxel plus cisplatin and 5-fluorouracil; PF, cisplatin plus 5-fluorouracil; TP, docetaxel plus cisplatin; GP, gemcitabine plus cisplatin; CPD, cumulative platinum dose.

**Supplementary Table S6**. Estimated 5-year survival outcomes of the 8 groups.

| Group | 5-year OS | 5-year DFS | 5-year DFFS | 5-year LRFFS |
| --- | --- | --- | --- | --- |
| 1 | 93.1% | 85.2% | 90.0% | 94.0% |
| 2 | 75.0% | 50.0% | 80.2% | 71.3% |
| 3 | 90.3% | 83.0% | 90.5% | 90.9% |
| 4 | 100% | 94.4% | 94.4% | 100% |
| 5 | 65.6% | 54.1% | 68.9% | 76.5% |
| 6 | 79.0% | 71.3% | 75.9% | 92.1% |
| 7 | 78.9% | 68.7% | 78.4% | 82.5% |
| 8 | 39.1% | 19.5% | 43.1% | 47.8% |

Abbreviations: OS, overall survival; DFS, disease-free survival; DFFS, distant failure-free survival; LRFFS, locoregional failure-free survival.

**Supplementary Table S7.** Results of multivariate survival analysis.

| Endpoint | Variable | HR (95% CI) | *P* value |
| --- | --- | --- | --- |
| OS | T category |  | 0.002 |
|  | T2 vs. T1 | 0.607 (0.298-1.237) | 0.169 |
|  | T3 vs. T1 | 0.692 (0.390-1.227) | 0.208 |
|  | T4 vs. T1 | 1.111 (0.616-2.005) | 0.727 |
|  | N category |  | < 0.0001 |
|  | N1 vs. N0 | 4.353 (0.597-31.759) | 0.147 |
|  | N2 vs. N0 | 6.855 (0.937-50.170) | 0.058 |
|  | N3 vs. N0 | 10.806 (1.465-79.719) | 0.02 |
|  | Risk group |  | < 0.0001 |
|  | 2 vs. 1 | 2.593 (0.801-8.390) | 0.112 |
|  | 3 vs. 1 | 1.493 (0.732-3.044) | 0.271 |
|  | 4 vs. 1 | NA | 0.947 |
|  | 5 vs. 1 | 3.158 (1.482-6.729) | 0.003 |
|  | 6 vs. 1 | 2.617 (1.286-5.325) | 0.008 |
|  | 7 vs. 1 | 2.553 (1.123-5.800) | 0.025 |
|  | 8 vs. 1 | 9.265 (4.438-19.343) | < 0.0001 |
| DFS | N category |  | 0.005 |
|  | N1 vs. N0 | 6.773 (0.937-48.970) | 0.058 |
|  | N2 vs. N0 | 8.821 (1.215-64.047) | 0.031 |
|  | N3 vs. N0 | 11.764 (1.613-85.801) | 0.015 |
|  | Risk group |  | < 0.0001 |
|  | 2 vs. 1 | 3.437 (1.325-8.914) | 0.011 |
|  | 3 vs. 1 | 1.254 (0.694-2.268) | 0.454 |
|  | 4 vs. 1 | 0.314 (0.041-2.378) | 0.262 |
|  | 5 vs. 1 | 3.113 (1.661-5.834) | < 0.0001 |
|  | 6 vs. 1 | 2.049 (1.120-3.749) | 0.02 |
|  | 7 vs. 1 | 2.149 (1.067-4.328) | 0.032 |
|  | 8 vs. 1 | 8.388 (4.511-15.599) | < 0.0001 |
| DFFS | Risk group |  | < 0.0001 |
|  | 2 vs. 1 | 1.244 (0.266-5.805) | 0.781 |
|  | 3 vs. 1 | 0.957 (0.449-2.038) | 0.909 |
|  | 4 vs. 1 | 0.561 (0.07-4.493) | 0.586 |
|  | 5 vs. 1 | 2.491 (1.132-5.483) | 0.023 |
|  | 6 vs. 1 | 2.282 (1.092-4.768) | 0.028 |
|  | 7 vs. 1 | 2.141 (0.906-5.061) | 0.083 |
|  | 8 vs. 1 | 6.421 (2.940-14.021) | < 0.0001 |
| LRFFS | Age (≤ 43 vs. > 43y) | 0.628 (0.402-0.981) | 0.041 |
|  | T category |  | 0.003 |
|  | T2 vs. T1 | 0.758 (0.233-2.469) | 0.645 |
|  | T3 vs. T1 | 1.810 (0.568-5.768) | 0.316 |
|  | T4 vs. T1 | 6.832 (1.782-26.182) | 0.005 |
|  | N category |  | 0.04 |
|  | N1 vs. N0 | 2.993 (0.403-22.236) | 0.284 |
|  | N2 vs. N0 | 3.673 (0.487-27.709) | 0.207 |
|  | N3 vs. N0 | 8.471 (1.051-68.264) | 0.045 |
|  | Risk group |  | < 0.0001 |
|  | 2 vs. 1 | 4.638 (1.145-18.792) | 0.032 |
|  | 3 vs. 1 | 1.583 (0.633-3.961) | 0.326 |
|  | 4 vs. 1 | NA | 0.962 |
|  | 5 vs. 1 | 5.206 (1.982-13.672) | 0.001 |
|  | 6 vs. 1 | 1.669 (0.610-4.570) | 0.319 |
|  | 7 vs. 1 | 2.804 (0.955-8.233) | 0.061 |
|  | 8 vs. 1 | 12.440 (4.766-32.467) | < 0.0001 |

Abbreviations: OS, overall survival; DFS, disease-free survival; DFFS, distant failure-free survival; LRFFS, locoregional failure-free survival; HR, hazard ratio; CI, confidence interval; NA, not assessed.

*P* values were calculated using an adjusted Cox Proportional hazards model with backward elimination and the following variables including gender, age, smoking, drinking, T category, N category, overall stage, induction chemotherapy regimen, cumulative platinum dose and risk group.

**Supplementary Table S8.** Estimated 5-year survival outcomes of different RPA staging.

|  |  | 5-year OS | 5-year DFS | 5-year DFFS | 5-year LRFFS |
| --- | --- | --- | --- | --- | --- |
| RPA1 | I | 91.80% | 83.50% | 89.70% | 90.80% |
|  | II | 85.70% | 77.90% | 86.70% | 89.30% |
|  | III | 66.90% | 58.00% | 67.10% | 85.50% |
|  | IV | 39.10% | 19.50% | 43.10% | 47.80% |
| RPA2 | I | 93.80% | 86.50% | 90.90% | 93.40% |
|  | II | 79.80% | 71.30% | 80.80% | 86.60% |
|  | III | 79.80% | 63.40% | 82.20% | 75.60% |
|  | IV | 60.80% | 50.80% | 62.30% | 79.90% |
| TNM staging | II | 91.20% | 79.20% | 87.50% | 89.40% |
|  | III | 87.20% | 79.10% | 86.00% | 89.70% |
|  | IV | 72.80% | 62.70% | 74.20% | 82.70% |

Abbreviations: OS, overall survival; DFS, disease-free survival; DFFS, disease failure-free survival; LRFFS, locoregional failure-free survival.

**Supplementary Table S9.** C-index of different staging systems.

| Endpoints | C-index | | |
| --- | --- | --- | --- |
|  | RPA1 | RPA2 | TNM |
| 5-year OS | 0.763 (0.714-0.812) | 0.735 (0.684-0.786) | 0.677 (0.604-0.749) |
| 5-year DFS | 0.731 (0.684-0.778) | 0.698 (0.650-0.747) | 0.631 (0.565-0.696) |
| 5-year DFFS | 0.744 (0.687-0.801) | 0.717 (0.656-0.777) | 0.643 (0.563-0.722) |
| 5-year LRFFS | 0.697 (0.618-0.775) | 0.649 (0.578-0.721) | 0.599 (0.499-0.698) |

Abbreviations: OS, overall survival; DFS, disease-free survival; DFFS, distant failure-free survival; LRFFS, locoregional failure-free survival.

**Supplementary Table S10**. Thresholds of Pre-IC DNA and RR identified in 100 bootstrap analysis.

| Bootstrap | Sample size | Pre-IC DNA | RR | Bootstrap | Sample size | Pre-IC DNA | RR |
| --- | --- | --- | --- | --- | --- | --- | --- |
| Bootstrap 1 | 395 | 11200 | 96.7% | Bootstrap 46 | 392 | 19400 | 95.13% |
| Bootstrap 2 | 408 | 15750 | 95.13% | Bootstrap 47 | 402 | 15050 | 96.7% |
| Bootstrap 3 | 397 | 9550 | 96.7% | Bootstrap 48 | 403 | 36500 | 96.3% |
| Bootstrap 4 | 404 | 14850 | 96.7% | Bootstrap 49 | 397 | 19650 | 95.13% |
| Bootstrap 5 | 389 | 15100 | 95.7% | Bootstrap 50 | 401 | 7430 | 95.13% |
| Bootstrap 6 | 406 | 16500 | 95.13% | Bootstrap 51 | 382 | 18500 | 95.2% |
| Bootstrap 7 | 393 | 50550 | 96.7% | Bootstrap 52 | 398 | 11250 | 95.13% |
| Bootstrap 8 | 391 | 16200 | 96.2% | Bootstrap 53 | 402 | 15750 | 95.13% |
| Bootstrap 9 | 408 | 7430 | 95.13% | Bootstrap 54 | 392 | 9750 | 93.51% |
| Bootstrap 10 | 396 | 15650 | 95.0% | Bootstrap 55 | 395 | 36000 | 96.24% |
| Bootstrap 11 | 390 | 36000 | 95.13% | Bootstrap 56 | 407 | 21600 | 95.8% |
| Bootstrap 12 | 391 | 11200 | 96.7% | Bootstrap 57 | 395 | 15000 | 89.32% |
| Bootstrap 13 | 404 | 16450 | 95.13% | Bootstrap 58 | 388 | 15050 | 89.32% |
| Bootstrap 14 | 409 | 7430 | 96.7% | Bootstrap 59 | 396 | 15050 | 95.13% |
| Bootstrap 15 | 381 | 35700 | 96.7% | Bootstrap 60 | 389 | 15050 | 96.3% |
| Bootstrap 16 | 404 | 4850 | 95.8% | Bootstrap 61 | 395 | 7295 | 95.13% |
| Bootstrap 17 | 392 | 20450 | 96.3% | Bootstrap 62 | 399 | 16500 | 96.3% |
| Bootstrap 18 | 381 | 15650 | 95.13% | Bootstrap 63 | 396 | 7430 | 95.13% |
| Bootstrap 19 | 396 | 16500 | 90.87% | Bootstrap 64 | 389 | 14950 | 96.7% |
| Bootstrap 20 | 395 | 15750 | 96.3% | Bootstrap 65 | 393 | 15050 | 95.4% |
| Bootstrap 21 | 404 | 36000 | 95.13% | Bootstrap 66 | 404 | 16000 | 90.87% |
| Bootstrap 22 | 394 | 12400 | 95.13% | Bootstrap 67 | 385 | 15750 | 96.7% |
| Bootstrap 23 | 391 | 17350 | 95.13% | Bootstrap 68 | 399 | 7430 | 95.13% |
| Bootstrap 24 | 388 | 15050 | 96.3% | Bootstrap 69 | 397 | 19650 | 95.2% |
| Bootstrap 25 | 395 | 11200 | 95.0% | Bootstrap 70 | 401 | 11400 | 90.87% |
| Bootstrap 26 | 386 | 12550 | 96.3% | Bootstrap 71 | 404 | 16200 | 95.0% |
| Bootstrap 27 | 401 | 9750 | 95.8% | Bootstrap 72 | 384 | 4890 | 95.13% |
| Bootstrap 28 | 399 | 15050 | 96.3% | Bootstrap 73 | 401 | 36000 | 95.04% |
| Bootstrap 29 | 405 | 11200 | 95.13% | Bootstrap 74 | 386 | 4310 | 95.13% |
| Bootstrap 30 | 390 | 7545 | 95.2% | Bootstrap 75 | 411 | 16500 | 95.13% |
| Bootstrap 31 | 380 | 18500 | 95.72% | Bootstrap 76 | 389 | 41650 | 95.04% |
| Bootstrap 32 | 400 | 21600 | 96.3% | Bootstrap 77 | 384 | 18900 | 96.7% |
| Bootstrap 33 | 406 | 3715 | 96.7% | Bootstrap 78 | 389 | 16500 | 96.7% |
| Bootstrap 34 | 402 | 16400 | 91.0% | Bootstrap 79 | 372 | 15100 | 96.3% |
| Bootstrap 35 | 397 | 11200 | 90.9% | Bootstrap 80 | 381 | 7250 | 95.2% |
| Bootstrap 36 | 410 | 15050 | 95.13% | Bootstrap 81 | 405 | 4670 | 95.13% |
| Bootstrap 37 | 392 | 18650 | 96.3% | Bootstrap 82 | 401 | 8395 | 95.13% |
| Bootstrap 38 | 401 | 12500 | 95.13% | Bootstrap 83 | 395 | 17350 | 95.13% |
| Bootstrap 39 | 389 | 8900 | 95.13% | Bootstrap 84 | 414 | 7585 | 94.4% |
| Bootstrap 40 | 400 | 15750 | 95.8% | Bootstrap 85 | 395 | 11200 | 95.13% |
| Bootstrap 41 | 401 | 15750 | 95.13% | Bootstrap 86 | 389 | 16500 | 96.3% |
| Bootstrap 42 | 391 | 16000 | 90.87% | Bootstrap 87 | 387 | 19350 | 95.72% |
| Bootstrap 43 | 398 | 14950 | 95.13% | Bootstrap 88 | 397 | 19400 | 95.13% |
| Bootstrap 44 | 408 | 18350 | 95.7% | Bootstrap 89 | 404 | 8395 | 95.8% |
| Bootstrap 45 | 393 | 41500 | 95.04% | Bootstrap 90 | 393 | 15650 | 95.13% |
| Bootstrap 91 | 391 | 15600 | 95.8% | Bootstrap 96 | 398 | 11200 | 96.7% |
| Bootstrap 92 | 396 | 15400 | 95.72% | Bootstrap 97 | 393 | 16850 | 95.72% |
| Bootstrap 93 | 397 | 35900 | 95.13% | Bootstrap 98 | 387 | 7430 | 96.3% |
| Bootstrap 94 | 392 | 7430 | 95.13% | Bootstrap 99 | 406 | 16800 | 95.13% |
| Bootstrap 95 | 387 | 4895 | 91.03% | Bootstrap 100 | 378 | 16500 | 95.13% |
| **Average** | **395.5** | **16060** | **95.163%** |  |  |  |  |

Abbreviations: Pre-IC DNA, plasma EBV DNA before induction chemotherapy; RR, remission rate.

**Supplementary Table S11.** AUC of all bootstrap analysis.

| AUC | 5-year OS | | | 5-year DFS | | | 5-year DFFS | | | 5-year LRFFS | | |
| --- | --- | --- | --- | --- | --- | --- | --- | --- | --- | --- | --- | --- |
|  | TNM | RPA1 | RPA2 | TNM | RPA1 | RPA2 | TNM | RPA1 | RPA2 | TNM | RPA1 | RPA2 |
| Bootstrap1 | 0.611 | 0.74 | 0.712 | 0.616 | 0.703 | 0.691 | 0.583 | 0.707 | 0.679 | 0.572 | 0.64 | 0.618 |
| Bootstrap2 | 0.604 | 0.727 | 0.692 | 0.576 | 0.676 | 0.686 | 0.593 | 0.683 | 0.694 | 0.565 | 0.623 | 0.658 |
| Bootstrap3 | 0.628 | 0.706 | 0.694 | 0.622 | 0.711 | 0.696 | 0.634 | 0.691 | 0.672 | 0.601 | 0.679 | 0.677 |
| Bootstrap4 | 0.672 | 0.758 | 0.714 | 0.639 | 0.724 | 0.689 | 0.635 | 0.754 | 0.709 | 0.6 | 0.658 | 0.636 |
| Bootstrap5 | 0.628 | 0.707 | 0.692 | 0.628 | 0.675 | 0.669 | 0.634 | 0.681 | 0.652 | 0.593 | 0.654 | 0.647 |
| Bootstrap6 | 0.642 | 0.747 | 0.716 | 0.624 | 0.694 | 0.698 | 0.622 | 0.722 | 0.69 | 0.591 | 0.653 | 0.652 |
| Bootstrap7 | 0.604 | 0.686 | 0.682 | 0.603 | 0.665 | 0.647 | 0.587 | 0.673 | 0.641 | 0.601 | 0.619 | 0.624 |
| Bootstrap8 | 0.603 | 0.723 | 0.708 | 0.621 | 0.716 | 0.692 | 0.623 | 0.718 | 0.708 | 0.59 | 0.69 | 0.636 |
| Bootstrap9 | 0.628 | 0.744 | 0.688 | 0.581 | 0.711 | 0.682 | 0.574 | 0.708 | 0.666 | 0.524 | 0.657 | 0.611 |
| Bootstrap10 | 0.651 | 0.772 | 0.734 | 0.616 | 0.734 | 0.694 | 0.61 | 0.715 | 0.682 | 0.582 | 0.707 | 0.621 |
| Average | 0.627 | 0.731 | 0.703 | 0.613 | 0.701 | 0.684 | 0.61 | 0.705 | 0.679 | 0.582 | 0.658 | 0.638 |

Abbreviations: AUC, area under curve; OS, overall survival; DFS, disease-free survival; DFFS, distant failure-free survival; LRFFS, locoregional relapse-free survival.

**Supplementary Table S12.** C-indexes of all bootstrap analysis.

| C-index | 5-year OS | | | 5-year DFS | | | 5-year DFFS | | | 5-year LRFFS | | |
| --- | --- | --- | --- | --- | --- | --- | --- | --- | --- | --- | --- | --- |
|  | TNM | RPA1 | RPA2 | TNM | RPA1 | RPA2 | TNM | RPA1 | RPA2 | TNM | RPA1 | RPA2 |
| Bootstrap1 | 0.674  (0.6-0.749) | 0.771  (0.721-0.822) | 0.749  (0.696-0.801) | 0.664  (0.6-0.729) | 0.744  (0.697-0.791) | 0.724  (0.675-0.773) | 0.64  (0.56-0.72) | 0.752  (0.695-0.808) | 0.722  (0.66-0.784) | 0.606  (0.497-0.715) | 0.7  (0.613-0.787) | 0.679  (0.599-0.759) |
| Bootstrap2 | 0.648  (0.565-0.731) | 0.744  (0.692-0.795) | 0.729  (0.669-0.788) | 0.612  (0.538-0.685) | 0.715  (0.664-0.766) | 0.71  (0.655-0.766) | 0.639  (0.545-0.732) | 0.727  (0.662-0.792) | 0.715  (0.644-0.786) | 0.597  (0.492-0.703) | 0.705  (0.627-0.784) | 0.671  (0.581-0.760) |
| Bootstrap3 | 0.674  (0.597-0.751) | 0.743  (0.688-0.797) | 0.725  (0.672-0.778) | 0.673  (0.605-0.741) | 0.752  (0.706-0.798) | 0.726  (0.680-0.772) | 0.711  (0.631-0.792) | 0.734  (0.677-0.791) | 0.705  (0.649-0.761) | 0.655  (0.55-0.76) | 0.741  (0.667-0.816) | 0.73  (0.665-0.794) |
| Bootstrap4 | 0.741  (0.675-0.807) | 0.776  (0.733-0.819) | 0.733  (0.685-0.781) | 0.686  (0.623-0.748) | 0.756  (0.714-0.799) | 0.714  (0.668-0.76) | 0.701  (0.628-0.773) | 0.791  (0.745-0.838) | 0.745  (0.691-0.799) | 0.639  (0.535-0.743) | 0.717  (0.639-0.795) | 0.687  (0.613-0.761) |
| Bootstrap5 | 0.678  (0.603-0.753) | 0.732  (0.676-0.788) | 0.728  (0.674-0.782) | 0.669  (0.603-0.736) | 0.709  (0.656-0.762) | 0.7  (0.65-0.751) | 0.681  (0.603-0.759) | 0.706  (0.643-0.77) | 0.676  (0.611-0.741) | 0.646  (0.550-0.742) | 0.702  (0.622-0.782) | 0.692  (0.625-0.76) |
| Bootstrap6 | 0.72  (0.651-0.788) | 0.77  (0.72-0.82) | 0.75  (0.697-0.802) | 0.68  (0.617-0.743) | 0.725  (0.678-0.773) | 0.734  (0.687-0.781) | 0.685  (0.608-0.763) | 0.757  (0.701-0.813) | 0.737  (0.675-0.800) | 0.639  (0.545-0.734) | 0.695  (0.616-0.774) | 0.702  (0.637-0.767) |
| Bootstrap7 | 0.641  (0.57-0.712) | 0.722  (0.671-0.773) | 0.707  (0.656-0.758) | 0.631  (0.566-0.695) | 0.697  (0.648-0.747) | 0.673  (0.623-0.723) | 0.635  (0.556-0.713) | 0.713  (0.653-0.774) | 0.673  (0.609-0.736) | 0.636  (0.54-0.731) | 0.664  (0.584-0.745) | 0.669  (0.597-0.742) |
| Bootstrap8 | 0.666  (0.594-0.739) | 0.759  (0.708-0.81) | 0.747  (0.696-0.799) | 0.652  (0.586-0.717) | 0.753  (0.707-0.8) | 0.729  (0.68-0.779) | 0.665  (0.586-0.743) | 0.749  (0.692-0.806) | 0.742  (0.683-0.801) | 0.626  (0.533-0.719) | 0.736  (0.665-0.807) | 0.69  (0.617-0.764) |
| Bootstrap9 | 0.674  (0.602-0.747) | 0.77  (0.722-0.818) | 0.715  (0.664-0.767) | 0.593  (0.524-0.661) | 0.736  (0.689-0.784) | 0.698  (0.65-0.747) | 0.592  (0.507-0.677) | 0.733  (0.672-0.793) | 0.694  (0.63-0.758) | 0.522  (0.418-0.625) | 0.705  (0.624-0.786) | 0.663  (0.59-0.736) |
| Bootstrap10 | 0.741  (0.68-0.802) | 0.8  (0.759-0.842) | 0.765  (0.718-0.812) | 0.66  (0.596-0.723) | 0.757  (0.714-0.8) | 0.712  (0.663-0.76) | 0.668  (0.589-0.747) | 0.753  (0.695-0.811) | 0.719  (0.654-0.785) | 0.616  (0.514-0.718) | 0.737  (0.667-0.807) | 0.67  (0.598-0.741) |
| Average | 0.686 | 0.759 | 0.735 | 0.652 | 0.734 | 0.713 | 0.662 | 0.74 | 0.714 | 0.618 | 0.707 | 0.689 |

Abbreviations: OS, overall survival; DFS, disease-free survival; DFFS, distant failure-free survival; LRFFS, locoregional relapse-free survival.

Data in the parentheses were 95% confidence intervals.

**Supplementary Table S13.** Results of multivariate analysis among patients with stage III-IV_RPA1_.

| Endpoint | Variable | HR (95% CI) | *P* value |
| --- | --- | --- | --- |
| OS | CPD (≥ vs. < 160 mg/m^2^) | 0.484 (0.287-0.818) | 0.007 |
| DFS | CPD (≥ vs. < 160 mg/m^2^) | 0.625 (0.390-1.002) | 0.051 |
| DFFS | CPD (≥ vs. < 160 mg/m^2^) | 0.657 (0.373-1.156) | 0.145 |
| LRFFS | CPD (≥ vs. < 160 mg/m^2^) | 0.918 (0.424-1.985) | 0.827 |
|  | IC regimen (PF vs. TPF) | 2.923 (1.391-6.143) | 0.005 |
|  | IC regimen (TP vs. TPF) | 1.426 (0.659-3.085) | 0.367 |
|  | IC regimen (GP vs. TPF) | NA | 0.983 |

Abbreviations: OS, overall survival; DFS, disease-free survival; DFFS, distant failure-free survival; LRFFS, locoregional failure-free survival; HR, hazard ratio; CI, confidence interval; CPD, cumulative platinum dose.

*P* values were calculated using an adjusted Cox proportional hazard model with backward elimination and following factors were included in the model: age (> vs. ≤ 43y), gender (female vs. male), smoking (yes vs. no), drinking (yes vs. no), induction chemotherapy regimen (PF vs. TPF, TP vs. TPF, GP vs. TPF) and CPD (≥ vs. < 160 mg/m^2^).

**Supplementary Table S14.** Results of multivariate analysis among patients with stage I-II_RPA1_.

| Endpoint | Variable | HR (95% CI) | *P* value |
| --- | --- | --- | --- |
| OS | CPD (≥ vs. < 160 mg/m^2^) | 1.124 (0.549-2.299) | 0.749 |
| DFS | CPD (≥ vs. < 160 mg/m^2^) | 1.128 (0.706-1.801) | 0.615 |
|  | IC regimen (PF vs. TPF) | 0.674 (0.352-1.291) | 0.234 |
|  | IC regimen (TP vs. TPF) | 0.479 (0.295-0.778) | 0.003 |
|  | IC regimen (GP vs. TPF) | 0.748 (0.103-5.406) | 0.773 |
| DFFS | CPD (≥ vs. < 160 mg/m^2^) | 0.824 (0.381-1.783) | 0.623 |
|  | IC regimen (PF vs. TPF) | 0.613 (0.256-1.465) | 0.271 |
|  | IC regimen (TP vs. TPF) | 0.462 (0.246-0.869) | 0.017 |
|  | IC regimen (GP vs. TPF) | 1.277 (0.175-9.343) | 0.810 |
| LRFFS | CPD (≥ vs. < 160 mg/m^2^) | 2.368 (0.952-5.886) | 0.064 |
|  | Age (> vs. ≤ 43y) | 0.461 (0.241-0.884) | 0.02 |

Abbreviations: OS, overall survival; DFS, disease-free survival; DFFS, distant failure-free survival; LRFFS, locoregional failure-free survival; HR, hazard ratio; CI, confidence interval; CPD, cumulative platinum dose.

*P* values were calculated using an adjusted Cox proportional hazard model with backward elimination and following factors were included in the model: age (> vs. ≤ 43y), gender (female vs. male), smoking (yes vs. no), drinking (yes vs. no), induction chemotherapy regimen (PF vs. TPF, TP vs. TPF, GP vs. TPF) and CPD (≥ vs. < 160 mg/m^2^).
