# Supplementary material for: Prognostic Value of Regression Rate of Plasma EBV DNA After Induction Chemotherapy for Stage II-IVA Nasopharyngeal Carcinoma
Source: Front Oncol. 2021 Jul 15;11:689593. doi: 10.3389/fonc.2021.689593 (PMC8319726; doi:10.3389/fonc.2021.689593)

**Supplementary Figures**

**Supplementary Figure S1**. Flow diagram of patient inclusion. NPC, nasopharyngeal carcinoma; IMRT, intensity-modulated radiotherapy; IC, induction chemotherapy; CCRT, concurrent chemoradiotherapy; pre-IC DNA, pre-treatment Epstein-Barr virus DNA; post-IC DNA, plasma Epstein-Barr virus DNA after induction chemotherapy. ^#^The prescribed radiation dose of the gross tumor volume was ≥ 66Gy. ^*^Patients were staged according to the 8th edition of the International Union against Cancer/American Joint Committee on Cancer (UICC/AJCC) staging system.


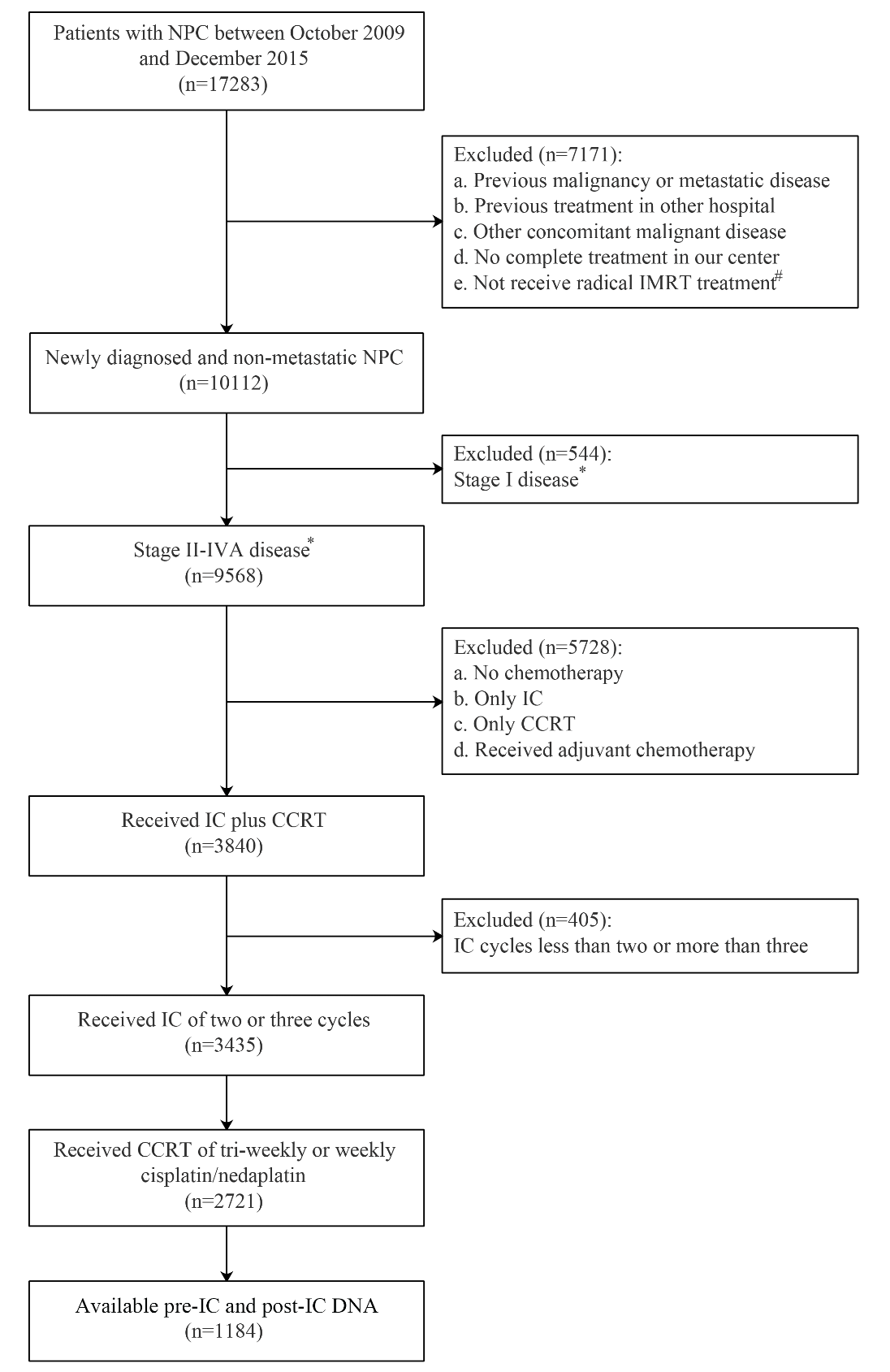


**Supplementary Figure S2**. Kaplan-Meier survival curves of (A) overall survival, (B) disease-free survival, (C) distant failure-free survival and (D) locoregional failure-free survival for the whole cohort.


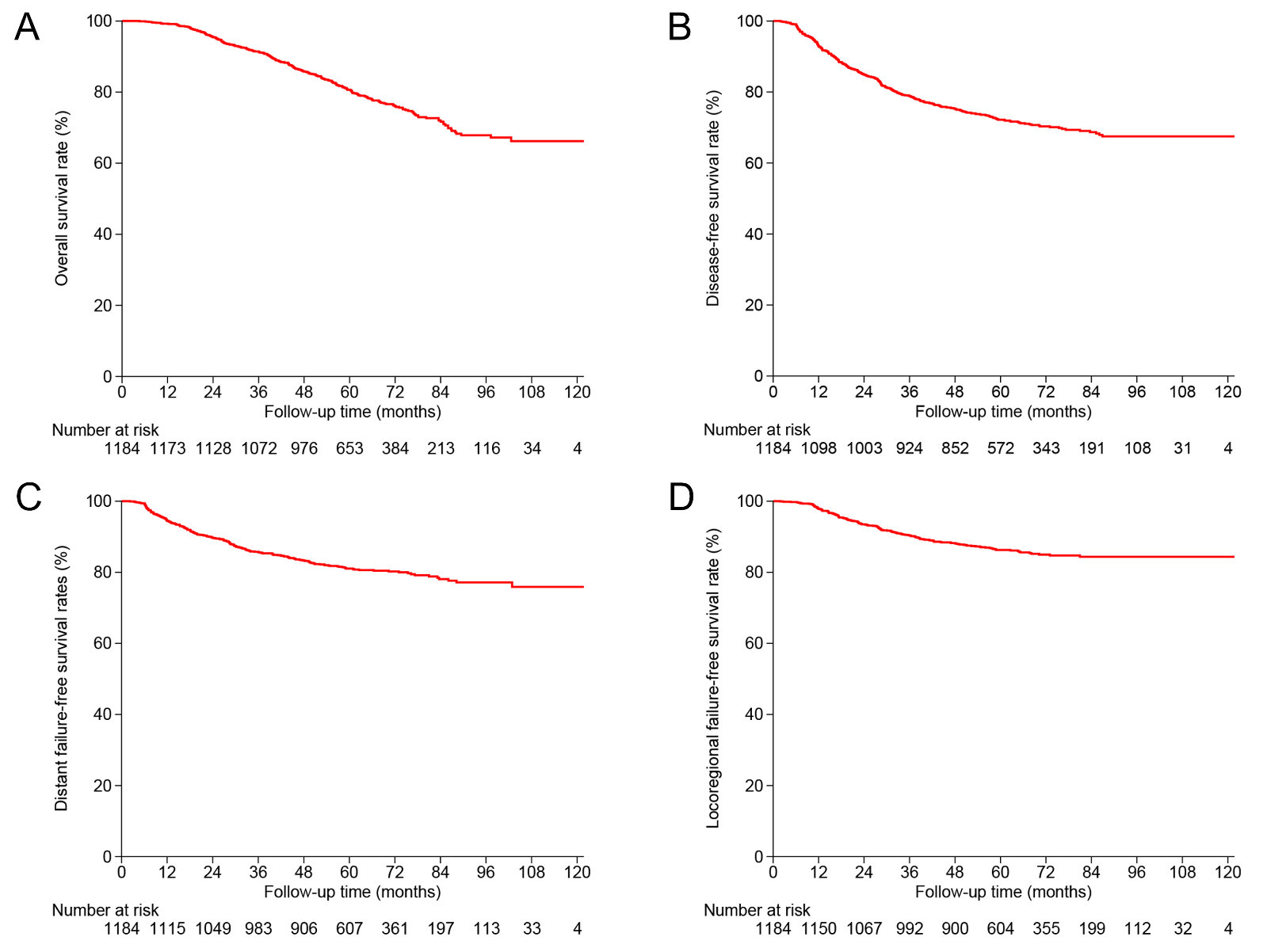


**Supplementary Figure S3**. Overall survival curves between (A) pre-IC DNA > and ≤ 16200 copies/ml in patients receiving 2 cycles, (B) pre-IC DNA > and ≤ 5520 copies/ml in patients receiving 3 cycles, (C) RR > and ≤ 95.127% in patients receiving 2 cycles and (D) RR > and ≤ 95.127% in patients receiving 3 cycles. Pre-IC DNA, pre-treatment Epstein-Barr virus DNA; RR, regression rate.


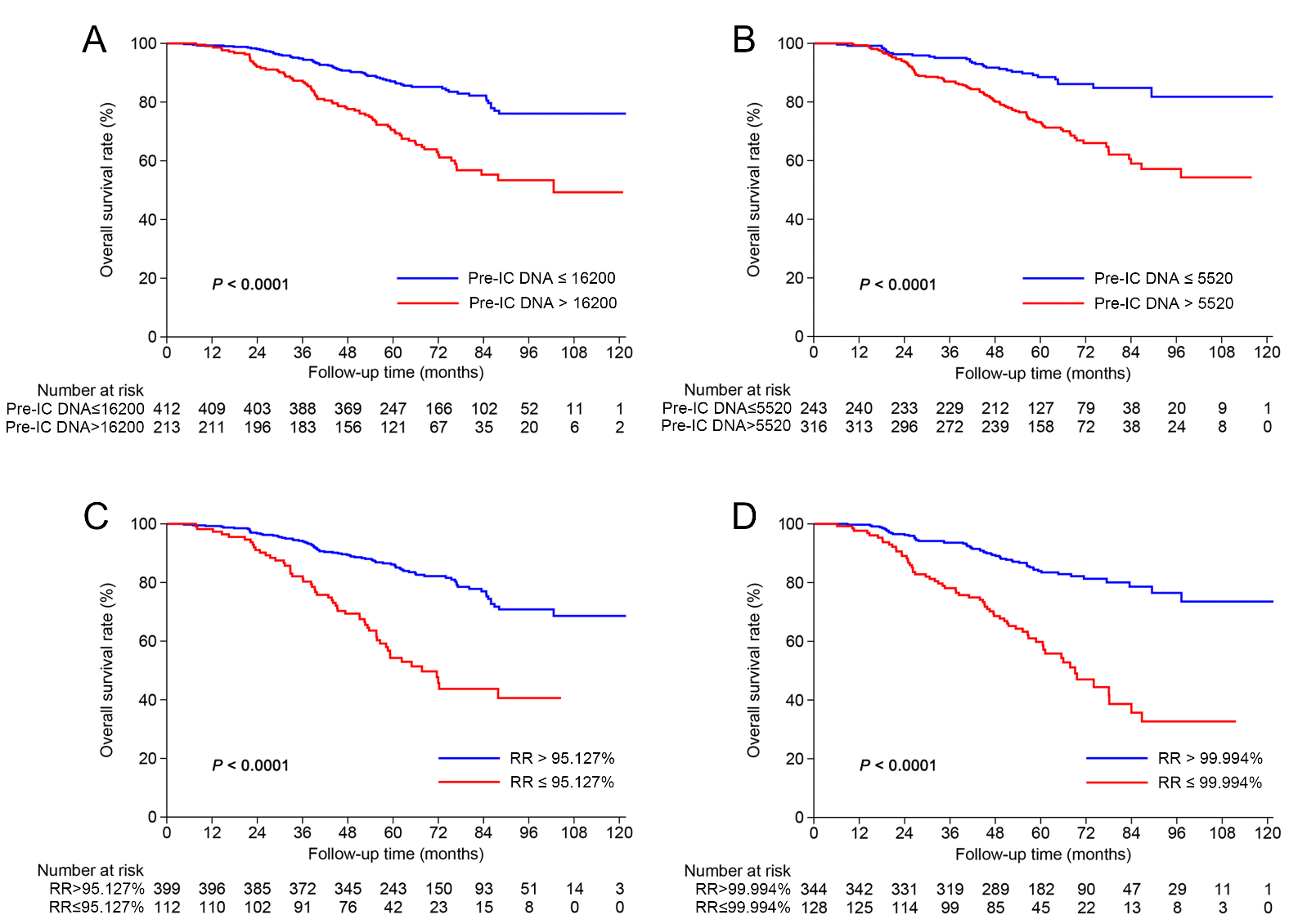


**Supplementary Figure S4**. Kaplan-Meier survival curves of (A) overall survival, (B) disease-free survival, (C) distant failure-free survival and (D) locoregional failure-free survival between patients with detectable and undetectable post-IC DNA. Post-IC DNA, plasma Epstein-Barr virus DNA after induction chemotherapy.


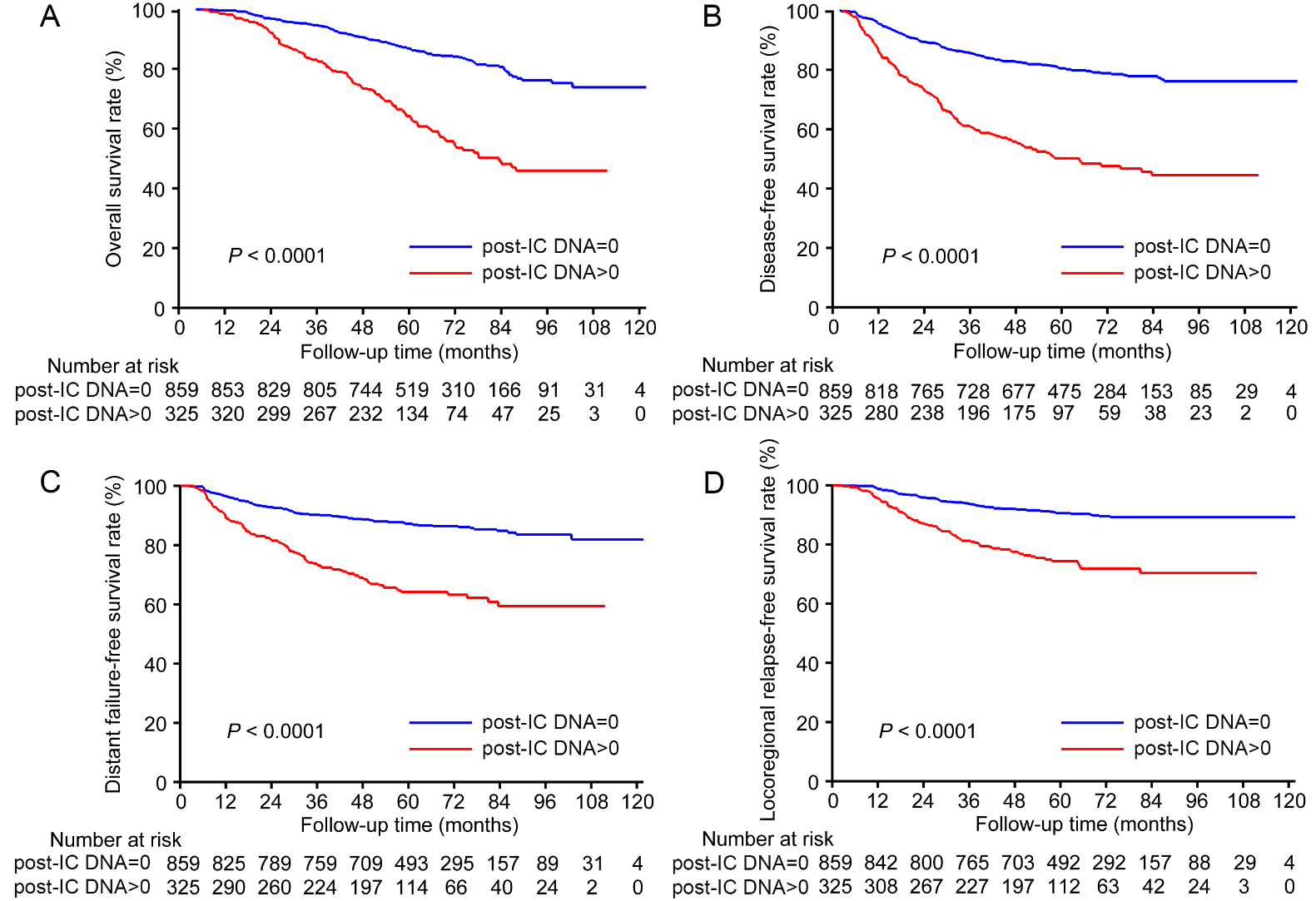


**Supplementary Figure S5**. Kaplan-Meier survival curves of (A) overall survival, (B) disease-free survival, (C) distant failure-free survival and (D) locoregional failure-free survival for the eight groups.


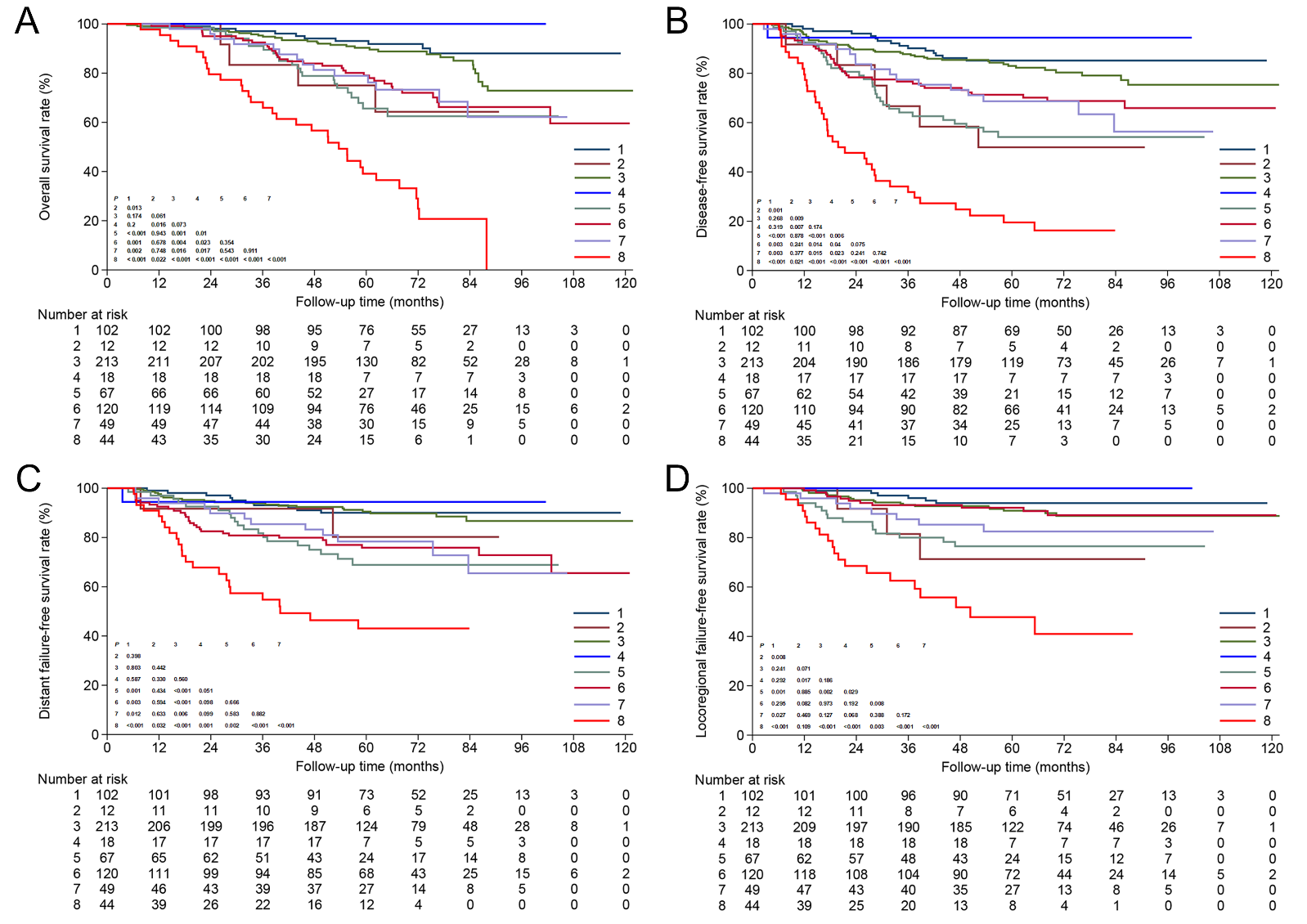


**Supplementary Figure S6**. Kaplan-Meier survival curves of (A) overall survival, (B) disease-free survival, (C) distant failure-free survival and (D) locoregional failure-free survival between different risk groups of RPA2 staging.


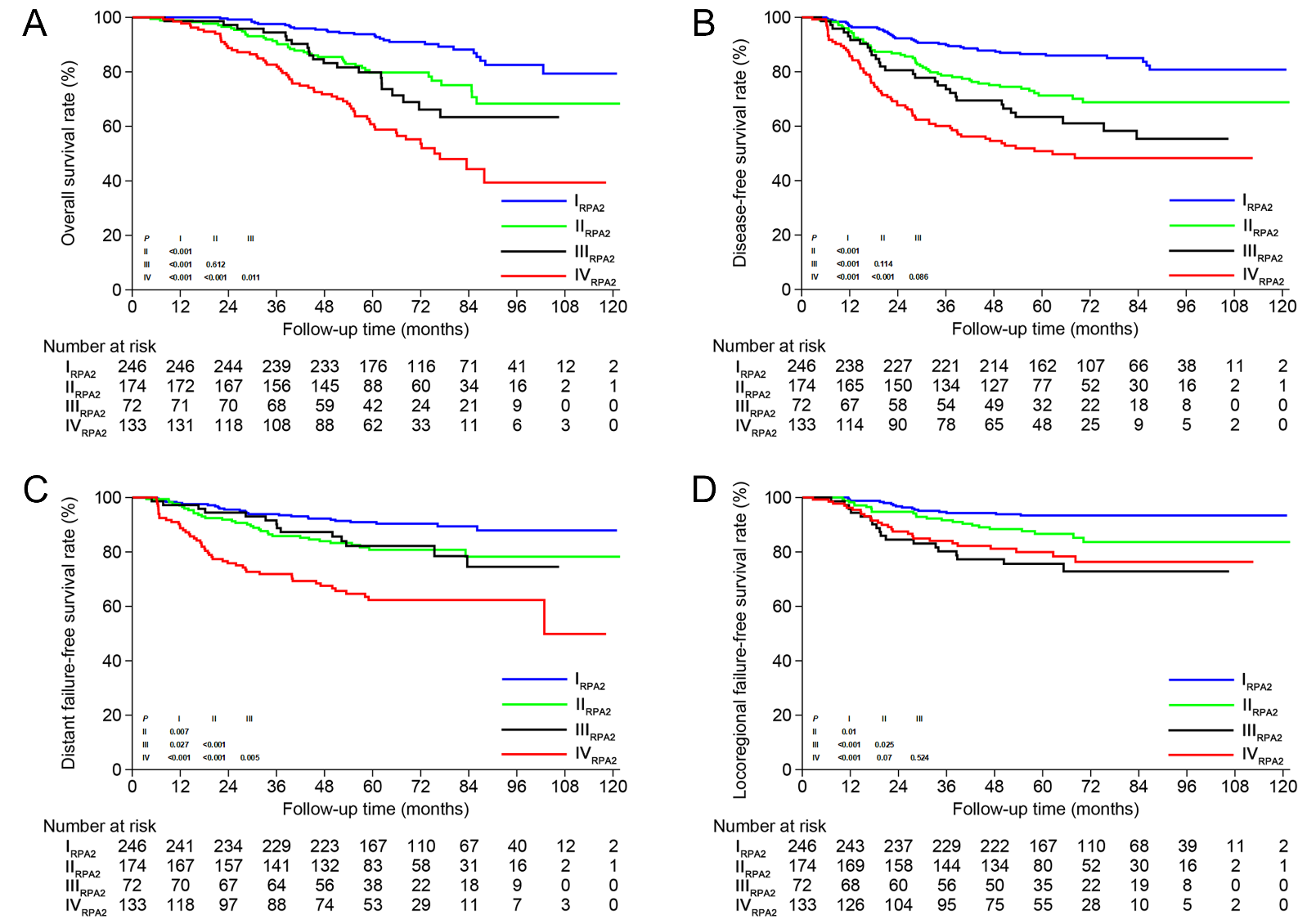


**Supplementary Figure S7**. Time-independent OS, DFS, DFFS and LRRFS ROCs of different staging in boostrap analysis. ROC, receiver operating curve; AUC, area under curve; OS, overall survival; DFS, disease-free survival; DFFS, distant failure-free survival; LRRFS, locoregional relapse-free survival.


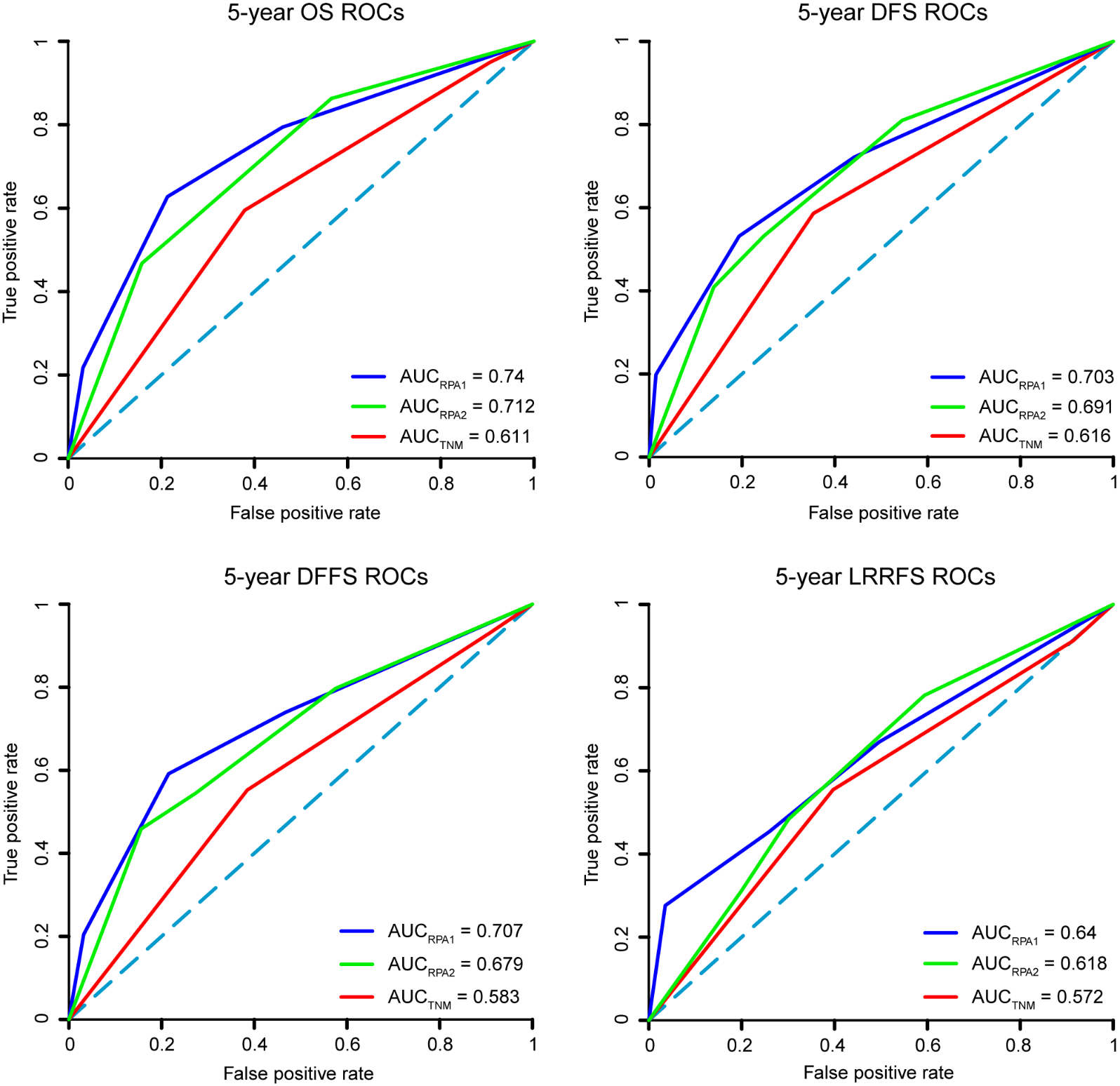


**Supplementary Figure S8**. Kaplan-Meier curves of overall survival between patients with a cumulative platinum dose < or ≥ 160 mg/m^2^ within different stages. (A) Stage II; (B) Stage III; (C) Stage IVA; (D) Stage II/III; (E) Stage II/IVA; (F) Stage III/IVA.


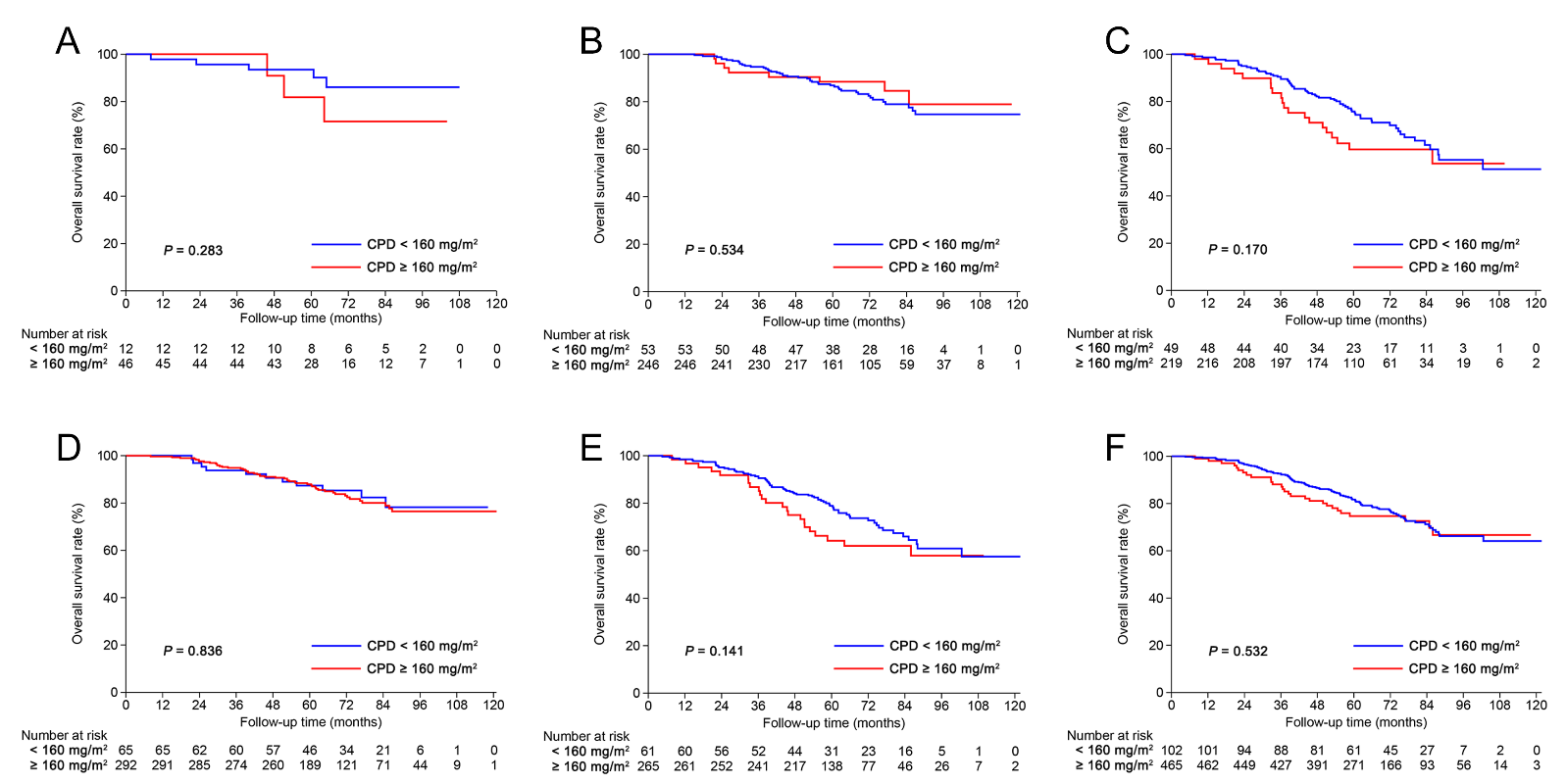


**Supplementary Figure S9**. Kaplan-Meier curves of (A) overall survival, (B) disease-free survival, (C) distant failure-free survival and (D) locoregional failure-free survival between patients with stage I-II_RPA1_ receiving a cumulative platinum dose < or ≥ 160 mg/m^2^.


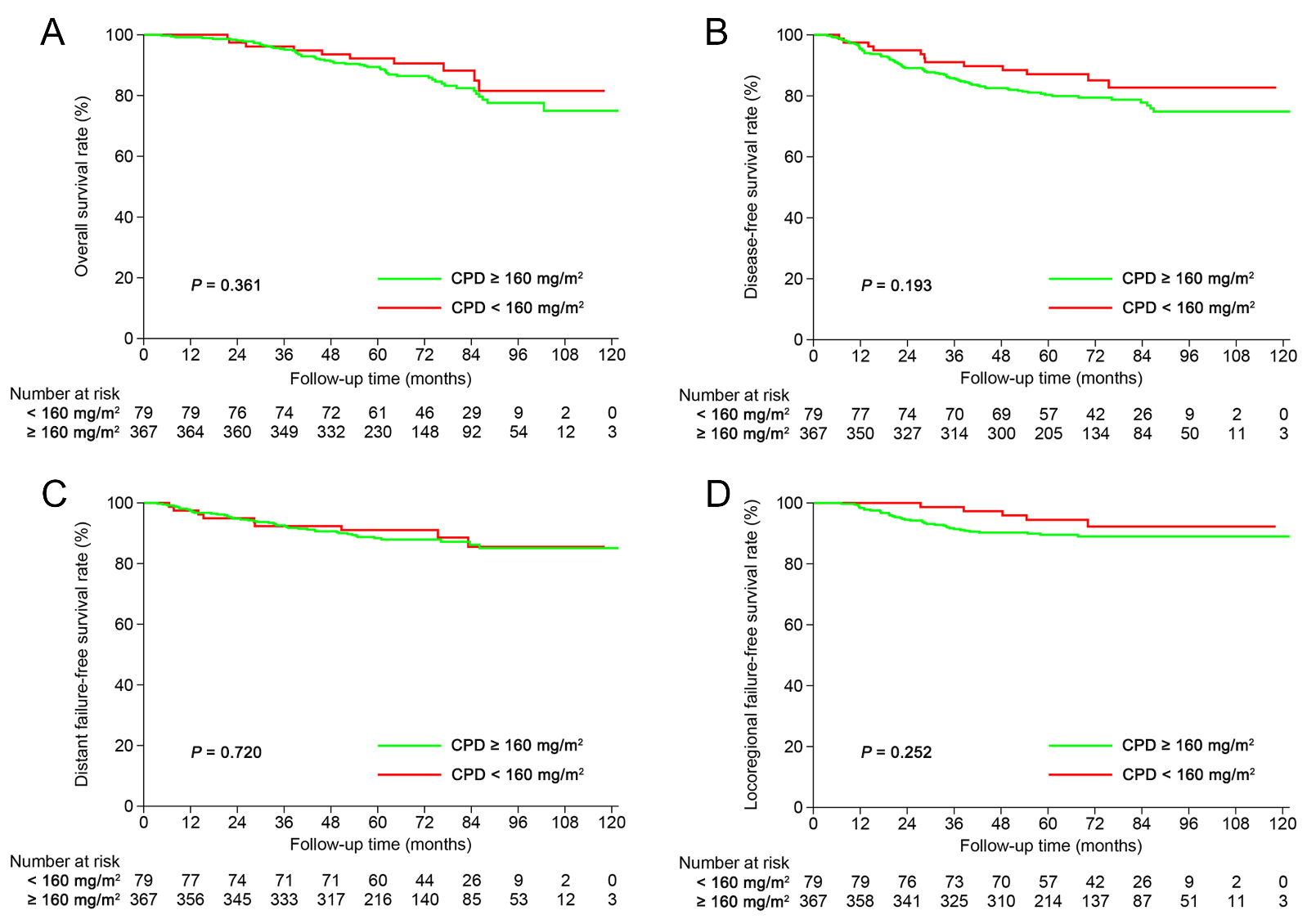

Supplement: Supplementary Figure 1 — Flow diagram of patient inclusion. NPC, nasopharyngeal carcinoma; IMRT, intensity-modulated radiotherapy; IC, induction chemotherapy; CCRT, concurrent chemoradiotherapy; pre-IC DNA, pre-treatment Epstein-Barr virus DNA; post-IC DNA, plasma Epstein-Barr virus DNA after induction chemotherapy. #The prescribed radiation dose of the gross tumor volume was ≥ 66Gy. *Patients were staged according to the 8th edition of the International Union against Cancer/American Joint Committee on Cancer (UICC/AJCC) staging system. [file DataSheet_1.docx]
